# Comparative Genomics of the Anopheline Glutathione S-Transferase Epsilon Cluster

**DOI:** 10.1371/journal.pone.0029237

**Published:** 2011-12-19

**Authors:** Constância Ayres, Pie Müller, Naomi Dyer, Craig Wilding, Daniel Rigden, Martin Donnelly

**Affiliations:** 1 Vector Group, Liverpool School of Tropical Medicine, Liverpool, United Kingdom; 2 Departamento de Entomologia, Centro de Pesquisas Aggeu Magalhães/FIOCRUZ, Recife, Pernambuco, Brasil; 3 Department of Medical Services and Diagnostics, Swiss Tropical & Public Health Institute, Basel, Switzerland; 4 University of Basel, Basel, Switzerland; 5 Institute of Integrative Biology, University of Liverpool, Liverpool, United Kingdom; University of California Riverside, United States of America

## Abstract

Enzymes of the glutathione S-transferase (GST) family play critical roles in detoxification of xenobiotics across many taxa. While GSTs are ubiquitous both in animals and plants, the GST epsilon class (GSTE) is insect-specific and has been associated with resistance to chemical insecticides. While both *Aedes aegypti* and *Anopheles gambiae* GSTE clusters consist of eight members, only four putative orthologs are identifiable between the species, suggesting independent expansions of the class in each lineage. We used a primer walking approach, sequencing almost the entire cluster from three *Anopheles* species (*An. stephensi*, *An. funestus* (both *Cellia* subgenus) and *An. plumbeus* (*Anopheles* subgenus)) and compared the sequences to putative orthologs in *An. gambiae* (*Cellia*) in an attempt to trace the evolution of the cluster within the subfamily Anophelinae. Furthermore, we measured transcript levels from the identified GSTE loci by real time reverse transcription PCR to determine if all genes were similarly transcribed at different life stages. Among the species investigated, gene order and orientation were similar with three exceptions: (i) *GSTE1* was absent in *An. plumbeus*; (ii) *GSTE2* is duplicated in *An. plumbeus* and (iii) an additional transcriptionally active pseudogene (*ψAsGSTE2*) was found in *An. stephensi*. Further statistical analysis and protein modelling gave evidence for positive selection on codons of the catalytic site in *GSTE5* albeit its origin seems to predate the introduction of chemical insecticides. Gene expression profiles revealed differences in expression pattern among genes at different life stages. With the exception of *GSTE1*, ψ*AsGSTE2* and *GSTE2b*, all *Anopheles* species studied share orthologs and hence we assume that GSTE expansion generally predates radiation into subgenera, though the presence of *GSTE1* may also suggest a recent duplication event in the Old World *Cellia* subgenus, instead of a secondary loss. The modifications of the catalytic site within GSTE5 may represent adaptations to new habitats.

## Introduction

Gene duplications are a major mechanism for acquisition of proteins with novel functions. Within the Insecta there are numerous examples where genes with putatively differing functions have arisen through serial duplication. Particularly noteworthy are the lineage-specific expansions in gene families associated with metabolism of toxic compounds [1]. One group of detoxification associated genes, the Glutathione S-Transferases (GSTs), appears to have undergone multiple independent radiations in the Diptera, *e.g.* in *Drosophila* [2] and Lepidoptera (*Bombyx mori*) [3]. This is a marked contrast with hymenopterans where in both *Apis* [4] and *Nasonia* [5] there is a relative paucity of GSTs. Particularly notable is the insect specific epsilon class (GSTE) in the Culicidae which has apparently undergone independent expansions in Anophelinae and Culicinae sub-families – whilst both *Aedes aegypti* and *Anopheles gambiae* contain eight GSTEs, only four putative orthologs (*GSTE2-4* and *GSTE8)* are identifiable, suggestive of independent gene duplication events [6]. It should be noted that while *GSTE8* is thought to be orthologous it is highly divergent (<29% amino acid identity) from the remaining seven genes and has been included in the family only due to its physical proximity to the other epsilon class members [7]. The multiple independent radiations of the GSTEs within the Diptera suggest that they are essential for the adaptation of dipterans to specific environmental pressures [4]. Interestingly, the non-dipterous, pea aphid *Acyrthosiphon pisum* and green peach aphid *Myzus persicae* appear to lack GSTEs [8]. Evidence for the role these genes play in the detoxification of xenobiotics comes from studies of resistance to the insecticide DDT. In *Ae. aegypti* and *An. gambiae* the orthologous GSTE2 proteins have both been shown to detoxify DDT through dehydrochlorination [9], [10]. Furthermore, quantitative genetic studies of a DDT-resistant *An. gambiae* colony localised a QTL around the GSTE cluster on chromosome 3R [11].

The divergence between the Culicinae and Anophelinae sub-families is an ancient one [12]. Maximum likelihood estimates based on protein-coding gene sequences place the *Anopheles* and *Aedes* split at between 145 and 200 Ma. Since the GSTE genes play such vital roles in detoxification it is of interest to know how this particular class has evolved, when the duplication events within *Anopheles* occurred and to attempt to relate this to aspects of the biology of the species. The *Anopheles* genus is split into seven subgenera (*Cellia*, *Anopheles*, *Nyssorhincus*, *Baimaia*, *Stethomyia*, *Kerteszia* and *Lophopodomyia*). The phylogenies within and between these subgenera have been the subject of much research ([12], [13], [14]). The *Cellia* subgenus has an Old World distribution while the *Anopheles* subgenus (*Anopheles* series) is cosmopolitan [13]. These subgenera are the largest within the *Anopheles* genus and are sister taxa that diverged between 90–106 Ma [12]. *Nyssorhincus* together with the last three subgenera have a neotropical distribution and *Baimaia* is restricted to Southeastern Asia. Within the *Cellia* subgenus the lineage including *An. funestus* (*Myzomyia* series) and *An. stephensi* (*Neocellia* series) is estimated to have diverged from that leading to *An. gambiae* (*Pyretophorous* series) around 36–80 Ma [12].

Whilst adult *Anopheles* of different species have broadly similar ecologies and food sources (mammalian and avian blood for females and nectar for males and females), larval ecological niches vary greatly from clean water to heavily polluted habitats, presenting larvae with widely differing toxic challenges. The genes which enable larvae to survive within such varied conditions, including the GSTE class, are likely targets of natural selection.

In the present study, we describe the diversification and expression pattern of GSTE in four different *Anopheles* species and address the following questions:

1. When did the duplication events occur and are they unique to specific lineages?
2. Is there evidence for natural selection acting upon the epsilon GSTs?
3. Do paralogous genes show the same patterns of expression in different life stages?

## Methods

### Mosquito specimens

Specimens from four species were used; *Anopheles funestus*, *An. gambiae, An. stephensi* and *An. plumbeus. An. funestus* specimens were collected in Agona Mansofo, southern Ghana in 2007 and in Ngelechom, near Tororo, eastern Uganda in 2008. *An. stephensi* (Beech colony originally from India) and *An. gambiae* (KISUMU, originating from western Kenya) specimens were obtained from the Liverpool School of Tropical Medicine (LSTM). Additional specimens of *An. stephensi* from Pakistan and Afghanistan [15] were included to confirm the presence of a putative pseudogene in field populations. Individuals of *An. plumbeus* were collected as larvae or pupae from tree holes at Stapleton Woods, Wirral, UK in 2008, and taken to the insectary of LSTM, where they were raised to adults (temperature 18°±2°C; relative humidity 60–80%; 12/12 h L/D). The typical *An. funestus* larval habitat is clean, lacustrine water. *An. gambiae* and *An. stephensi* are more catholic in their preferences with habitats varying between temporary (e.g. puddles) and more permanent (e.g. rice paddies) water bodies and have even been found in highly organically polluted breeding sites [16]. *An. plumbeus* is found only in the tannin-rich water in tree holes, typically full of rotting vegetation.

Species were identified morphologically and their status confirmed through PCR of the internal transcribed spacer of rDNA (ITS2). Total DNA was extracted from individual mosquitoes using the QIAGEN DNEasy extraction kit (Qiagen, Crawley, UK), according to the manufacturer's instructions. ITS2 primers targeting the ribosomal RNA 5.8S and the 28S [17], [18] were used to amplify fragments of approximately 560 bp, 600 bp, 840 bp and 337 bp in *An. gambiae* [19], *An. stephensi* [20], *An. funestus* [19] and *An. plumbeus* [21], respectively. PCR amplification was carried out in 50 µl reactions containing 2.5 µM MgCl_2_, 200 µM of each dNTP, 0.2 µM of each primer, 5 µl of 10x PCR buffer, 1 unit of *Taq* DNA polymerase (Bioline) and 10 ng template DNA. PCR reactions were incubated at 94°C for 5 min, followed by 35 cycles at 94°C for 1 min, 60°C for 30 s and 72°C for 30 s, with a final extension at 72°C for 7 min. Ten microliters of PCR products were run on a 1% agarose gel and visualized by ethidium bromide staining. The size of bands was estimated based on a 100 bp ladder (Bioline).

### Primer walking, gene cloning and DNA sequencing

Culicidae genome sequences were available only for *An. gambiae*, *Culex quinquefasciatus* and *Aedes aegypti* [22], [23], [24]. Therefore, we designed primers based upon the *An. gambiae* genome or on the consensus sequence between *An. gambiae* and *Ae. aegypti* to amplify genes from the GSTE cluster in the other taxa. Various primer combinations were used to amplify each GSTE gene and subsequently used in combination to amplify intergenic regions. Where primer combinations yielded large amplicons (>4.0 kbp) the Long Range PCR kit from QIAGEN (Crawley, UK) was used according to the manufacturer's instructions. Due to the high divergence of *GSTE8* from other epsilon class members, we did not attempt amplification of this gene.

PCR products were purified using QIAquick PCR purification kit or a QIAquick Gel Extraction Kit (Qiagen, Crawley, UK) if more than one band was present. Amplicons were cloned into a pGEMT-Easy plasmid (Promega, Southampton, UK) and sequenced using universal primers. An iterative primer walking approach was employed to obtain full-length bidirectional sequences by designing specific primers (PrimerSelect™, DNASTAR Inc), for each species based on sequences obtained in the previous sequencing round (i.e. forward primer at the 3′ end of the previous segments).

### Sequence analysis

After trimming vector regions, sequences were assembled using CodonCode Aligner 2.0.4 (default assembly criteria: 70% minimum percent identity and 25 bp minimum overlap length). FASTA files and predicted amino acid sequences of GSTE from *An. gambiae* and *Ae. aegypti* were downloaded from VectorBase (http://www.vectorbase.org/index.php). Sequences were aligned using the ClustalW algorithm (gap extension penalty: 1; gap initiation penalty: 3) in BioEdit 7.0 [25] and manually annotated by comparing obtained sequences against the *An. gambiae* template. For gene naming we followed the unified GST nomenclature proposed by Chelvanaygan *et al.* [26]. All sequences have been deposited in GenBank (for accession numbers see Supplementary Table S1). Sequences of *An. darlingi* (*Nyssorhyncus* sub-genus) for the tests of selection were kindly provided by Dr Ana Tereza Vasconcelos (Laboratório Nacional de Computação Científica, Petropolis, RJ, Brazil). The whole genome of *An. darlingi* is now available under the accession number ADMH00000000 (DDBJ/EMBL/GenBank). GSTE sequences from *Drosophila melanogaster* were downloaded from FlyBase (http://flybase.org/blast/).

In an attempt to identify putative regulatory elements we searched for motifs using two bioinformatics tools, MEME [27] and MAST [28] (http://meme.sdsc.edu/meme4/cite.html). Identification of conservative motifs within intergenic regions and 3′ untranslated regions (3′UTR) was done by: 1) comparing the different regions within the same species (species-specific motifs) and 2) comparing the same region across different species (locus-specific motifs).

### Structure modelling

Protein structure models were constructed for the paralogous *An. plumbeus* GSTE2 and GSTE2B sequences and for the *An. gambiae* GSTE5 protein. In each case, the single template used for model construction was the *An. gambiae* GSTE2 structure [29] (PDB code 2imk). The three target sequences share 77, 86 and 52% sequence identity, respectively, with the template. For each target, 10 models were generated and the final model was that with the best DOPE score [30]. PyMOL (http://www.pymol.org) was used for visualisation, manipulation and comparison of structures, and for production of structure figures.

### Gene trees and tests of selection

In addition to the *An. funestus*, *An. stephensi*, *An. plumbeus* and *An. gambiae* sequences described above, available *An. darlingi* (ADMH00000000) and *An. cracens* (GSTE2: Genbank GU128143.1, and GSTE4: Genbank DQ168030) sequences were used for construction of gene trees and tests of selection. GSTE protein sequences were inferred by translation, aligned using default settings in PRANKSTER [31], and back translated to make the nucleotide alignment. This approach results in an alignment of codons suitable for further analysis of codon selection.

Comparison of paralagous genes means that there are high level of sequence divergence and possible saturation of substitutions at synonymous sites, which could lead to an underestimation of the evolutionary distance between sequences and the number of synonymous substitutions. The number of synonymous substitutions per synonymous site (kS) was estimated using DNAsp [32] for all pairs of sequences. For paralogs it ranged from 0.5 to 5.5, (mean = 1.6, s.d. = 0.7). Orthologous genes had lower levels of kS (range 0.05–2.0 (mean = 1.1, s.d. = 0.4)). These moderate levels of saturation did not markedly affect tree topology. Phylogenetic trees based upon data from the third codon position, second codon position or all codon positions are topologically very similar (data not shown). This implies that despite the high estimated kS, the synonymous substitutions have not reached total saturation and a phylogenetic signal is retained. We therefore continued to use information from all sites, including synonymous sites, to infer trees and conduct tests of selection. Modeltest [33] suggested, based on Akaike Information Criterion, that the General Time Reversible substitution model with a gamma distribution of rates among sites (GTR + G) best described the dataset out of 88 candidate models. The GTR + G model was therefore used in maximum likelihood tree construction using PhyML online [34], with other parameters estimated from the data. 500 bootstrap replications were performed to assess the robustness of the branching.

To test the hypothesis of positive selection in GSTE genes we used the Codeml program within PAML v4.2 [35], [36]. Tests are based on comparing synonymous (dS) and non-synonymous (dN) substitution rates of the coding regions, with positive selection implied by dN/dS (ω) ratios >1. Three types of tests were applied using nested models: site models were used to test for variation in ω among sites [37], [38]; branch models [39], [40] were used to test for variation in ω among branches of the phylogeny and to search for positive selection in the lineage leading to *GSTE5*; and branch site models were used to test for sites under selection in individual branches of the tree [41], [42]. The relative likelihoods of contrasting models given the data were assessed using likelihood ratio tests (LRT). The statistic 2δ = 2[Ln*L*(M1) – Ln*L*(M2)] is χ^2^ distributed for nested models, with the number of degrees of freedom being the difference in the number of free parameters estimated by the two models. Calculations for all models were run three times. Sites under positive selection were identified using a Bayes Empirical Bayes (BEB) analysis [43].

Site tests were performed largely as described in [37], [38]. To detect sites under positive selection the likelihood of the data was compared using likelihood ratio tests under the following models: 1. Model 1a (neutral: ω≤1 at a proportion p_0_ of sites, ω_1_ = 1 at a proportion p_1_ of sites) was compared with model 2a (positive selection ω≤1 at p_0_ sites, ω_1_ = 1 at p_1_ sites and ω_2_≥1 at p_2_ sites). 2. Model 7 (beta) which has 10 site classes with ω≤1 with a beta distribution of ω among sites was compared with model 8 (beta and ω) which has 10 site classes, each at proportion p_0_ of sites with ω≤1 with a beta distribution of ω among sites, plus one site class at proportion p_1_ sites with ω_s_≥1. 3. Model 8 was compared with model 8a, which is similar to model 8 except that ω_s_ = 1.

For the branch tests, heterogeneity of ω amongst branches was tested by comparing branch model 0 (all branches constrained to have the same ω) with branch model 1, in which ω is estimated separately for each branch. The number of ω values estimated in branch model 1 is determined by the number of branches, which is 2n−3, where n is the number of sequences in the tree. GSTall contains 31 sequences and therefore 59 branches; GST no e6 pfd contains 28 sequences and therefore 53 branches. In branch model 1, in different replicates between three and five branches were found to have ω>1. We decided to focus on the *GSTE5* branch as the foreground branch because it has a relatively high dN of 0.11, ranked 4/59 estimated dNs. The other branches with ω>1 had low relatively dN ranked below 20/59, out of all the estimated dNs, and very low dS values, suggesting that their high estimated ω values may be a result of the high variability in ω due to the branches being very short, with low dS. We tested the hypothesis that the *GSTE5* branch has a higher ω than the other branches by comparing model 0 to strict model 2, in which the *GSTE5* branch has ω_1_, estimated independently from the other branches, and all other branches have ω≤1. We tested the hypothesis that the *GSTE5* branch is under positive selection as opposed to merely a relaxation of purifying selection by comparing strict model 2 with relaxed model 2, in which ω_1_ is constrained to 1 (i.e. neutral).

For the branch site tests [41], [42] we used “test 2” [42], which compares the likelihood of the models A1 and A2 outlined in Supplementary Table S2. Both models have four site classes and background and foreground branches. The null model A1 allows sites under purifying (negative) selection (0<ω<1) and under neutral evolution (ω_1_ = 1) in background branches and allows some sites on foreground branches to evolve neutrally (ω_2_ = 1). Model A2 differs only in that ω_2_ is freely estimated so that we test specifically for positive selection at sites in the foreground branch and not merely a relaxation of selective constraint.

To test the power and accuracy of test of selection in the site and branch tests, simulated datasets were generated using Evolver in the PAML suite [35], [36]. The data was simulated to resemble the GSTall data set: there were 31 taxa represented by 257 codons of data, using the *Anopheles gambiae* codon usage table. The 31 taxa were related by the same tree with the same branch lengths as the true dataset, and there were 4 site classes in the same proportions as estimated for the real data under branch site model A2, with the omega ratios in foreground and background branches being the same as estimated for the real data under either model A1, to test the rate of false positive detection of positive selection, or under model A2 to test the power and accuracy of site and branch tests and BEB detection of sites on the foreground branch under positive selection. 100 simulated datasets were used for each test. For model A2, simulations were performed with a foreground omega (ω_2_) in site classes 2a and 2b of 4, 9 and 999 to represent low, moderate and estimated values respectively. Simulated datasets were tested using the site and branch test in codeml under models A1 and A2, in the same way as the real data. To test the accuracy of the detection of sites under positive selection on the foreground branch (belonging to site class 2a or 2b) by BEB, the program PositiveSitesBS from the PAML suite [35], [36] was used to compare the sites actually simulated to be under positive selection as outputted by Evolver, and those found to be under positive selection by codeml for each dataset simulated under model A2. To test the effect of the level of divergence and possible saturation of substitutions on the power, accuracy and false positive rate, simulations were performed with branch lengths of half the length and double the length of the branches in the tree estimated for real data under branch site model A1 and branch site model A2 with ω_2_ = 9.

### Identification of 3′untranslated regions (UTR)

Rapid amplification of cDNA ends (RACE) was used to obtain 3′ UTRs of each GSTE gene. First strand cDNA synthesis was carried out using the 3′ RACE System (Invitrogen, Paisley, UK) according to the manufacturer's instructions. Conditions for nested PCR were optimized for each specific primer (equilibrating the PCR mixtures for 1 min at 80°C after setting up the reactions on ice, followed by 3 min at 94°C and 30 cycles of 94°C for 30 sec, 50 to 60°C for 30 sec and 72°C for 1 min, with a final extension at 72°C for 7 min). PCR was performed using the lock-docking oligo dT primer [44] and gene-specific primers (Primer sequences are given in Supplementary Table S3). To assess the potential role of regulatory sequences we searched for conserved 3′UTR regions across loci as described above (see Sequence Analysis) and microRNA (miRNA) target sites that might be involved in post-transcriptional regulation. Targets of all *An. gambiae* miRNA sequences listed in miRBAse [45] were predicted computationally. In total, 65 unique *An. gambiae* mature miRNA sequences served as input, including ten miRNAs cloned from *An. gambiae* [46], eight from *An. stephensi* [47] and 47 additional miRNA sequences identified from the *An. gambiae* genome through similarity to already known miRNA sequences. Since it is likely that the list of 65 miRNAs is not exhaustive, we additionally used a second input file of 147 miRNAs from *D. melanogaster* from which most miRNAs have been described. Experimentally determined GSTE 3′UTRs from *An. stephensi*, *An. plumbeus* and *An. funestus* served as input. For *An. gambiae*, GSTE 3′UTRs were not confirmed experimentally and instead intergenic 3′ sequences (maximum length 1 kbp) were utilised. Targets were predicted using miRanda 3.0 [48]; [49]. MiRNAs were first scanned against all 4,033 known *An. gambiae* 3′UTRs downloaded from Biomart (http://metazoa.ensembl.org/biomart) and since for many loci there is no experimentally determined UTR information, the region 1 kb upstream of all genes (No. of genes = 13,621). From this, an extreme value distribution (EVD) was computed representing the genomic background of miRanda scores following the model of Rehmsmeier et al. [50]. MiRNA-specific EVD profiles then served as ancillary input to MiRanda allowing computation of miRNA:potential-target *P*-values. Following identification of miRNA targets utilising *D. melanogaster* mature miRNA sequences, the *An. gambiae* genome sequence was subsequently searched for miRNA precursor sequences using MapMi (http://www.ebi.ac.uk/enright-srv/MapMi/index.html).

### Gene expression analysis

Quantitative reverse transcription (qRT-PCR) was used to measure gene expression levels of selected GSTEs in *An. funestus*, *An. gambiae* and *An. stephensi* in order to determine whether all GSTEs are transcriptionally active. The PCR protocol is described in Müller et al. [51]. An aliquot of 75 ng from each RNA pool served as template for making target specific cDNA by reverse transcription in a single multiplex assay, using the GenomeLab GeXP Start Kit (Beckman Coulter, High Wycombe, UK). For the RT reaction and subsequent PCR the gene-specific primers listed in Supplementary Table S3 were used.

## Results

### Gene organization and intron/exon structure

**Figure 1:**
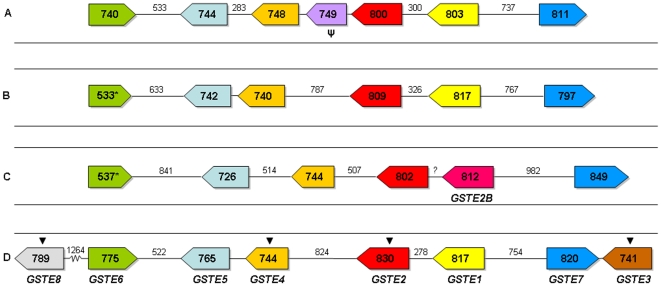
Comparison of the structure of GSTE clusters in the three *Anopheles species.* Transcriptional orientation of each GST gene is shown by an arrow. The size of each gene is indicated in the boxes and the intergenic region size is shown above the lines. A) *Anopheles stephensi*; B) *Anopheles funestus*; C) *Anopheles plumbeus* and D) *Anopheles gambiae*. * indicates that gene sequence is not complete. Arrows above the genes indicate orthologs with *Aedes aegypti*. *GSTE8* and *GSTE3* were not amplified in *An stephensi*, *An. funestus* and *An. plumbeus*.

**Figure 2:**
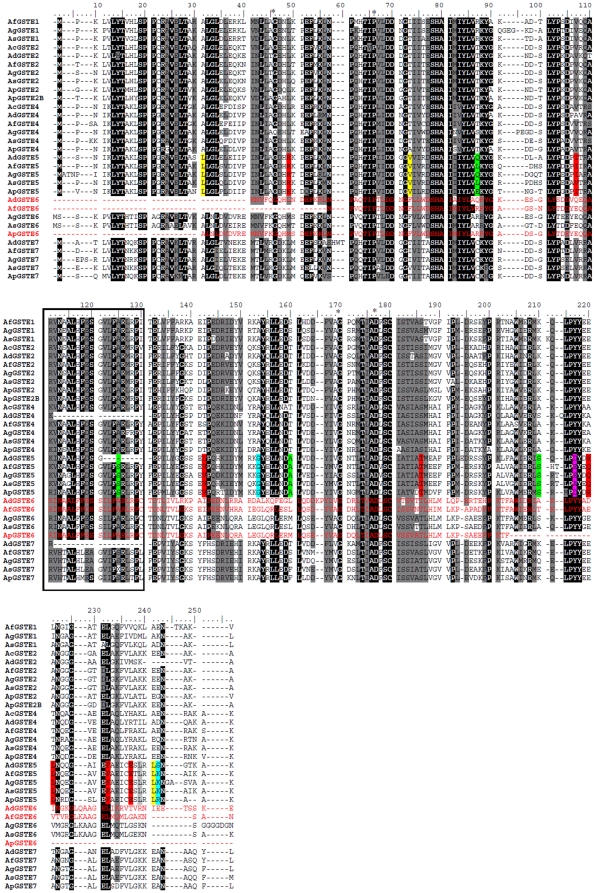
Alignment of amino acid residues of the GST epsilon class in *Anopheles* species. Residue numbering for each sequence is shown at the top. Conserved residues are shaded (>80%). The conserved region in the C-terminal domain is boxed. * represents amino acid highly conserved among GSTs. Sites under selection have been highlighted in colour. The three shorter sequences excluded from the second analysis are in red type. The highlighted sites were inferred by the Bayes Emperical Bayes method to have ω>1. The probability of the site being assigned to a class with ω>1 is indicated by the color of the shading: yellow: P>0.99 in both GSTall and GSTnoe6pfd; red: 0.95<P<0.99 in both GSTall and GSTnoe6pfd; green: 0.95<P<0.99 in GSTnoe6pfd only; blue: 0.95<P<0.99 in GSTall only; pink: 0.95<P<0.99 in GSTall, P>0.99 in GSTnoe6pfd; grey: P>0.99 in GSTall, 0.95<P<0.99 in GSTnoe6pfd.

**Figure 3:**
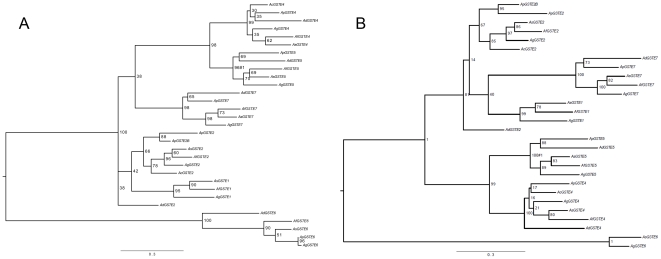
Phylogenetic relationship of *Anopheles* epsilon class GSTs. Maximum likelihood trees for epsilon class GSTs used in PAML analysis. Branch support is given as a percentage of 500 bootstrap replicates. A) For all available sequences and B) excluding truncated sequences for GSTE6 for *Anopheles funestus*, *An. plumbeus* and *An. darlingi*. The foreground branch used in the branch and branch-site models is marked #1. Note that while midpoint rooted trees are shown here for ease of reading; unrooted trees were used in PAML analysis.

In total, we could amplify and characterise six GSTE genes, i.e. *GSTE6*, *GSTE5*, *GSTE4*, *GSTE2*, *GSTE1* and *GSTE7*. While *An. plumbeus* lacked *GSTE1* all other GSTEs were found in all species studied (i.e. *An. gambiae*, *An. stephensi*, and *An. funestus*). *GSTE6* could only partially be characterized for *An. plumbeus* and *An. funestus*. We were also not successful in amplifying *GSTE3* from any species. All genes are arranged in the same way - order and orientation – and contain the same number of introns and exons as seen in *An. gambiae* (Fig. 1). High sequence variation in introns was observed between the four species (mean sequence identity = 0.274). Introns were small, ranging from 59–75 bp in *An. stephensi*, 61–83 bp in *An. funestus* and 60–105 bp in *An. plumbeus* (Table 1) and can be classified as phase 0 introns (*i.e*. the intron is between two codons), with the exception of the second intron in *GSTE7* and the *GSTE6* intron which can be classified as phase 1 introns (*i.e*. the intron is between the first and second nucleotide of the codon). In *An. plumbeus* an additional GSTE was found located between *GSTE2* and *GSTE7*. Its sequence was very similar to *ApGSTE2* (amino acid sequence identity 81.4% Supplementary Table S4; Fig. 2 and 3) and is therefore considered a duplicate *GSTE2* and named *ApGSTE2B*. The intergenic region between *GSTE4* and *GSTE2* in *An. stephensi*, a 975 bp long sequence, displayed an exon putatively orthologous to the second exon of *AsGSTE2*. This region is characterized by various premature stop codons and does not have an open reading frame, suggesting it is a pseudogene. Here, we will name it *ψAsGSTE2*. However, this sequence showed a high conservation level among individuals from Pakistan, Afghanistan and the Beech colony (Figure S1) and was shown to be transcribed (see 3′ RACE discussion below) suggesting that it may be a true gene or have a regulatory function.

**Table 1:** Variation in intron size and amino acid (AA) sequences for *An. gambiae*, *An. funestus*, *An. stephensi* and *An. plumbeus.*

Genes/introns	*An. gambiae*	Putative AA sequence	*An. stephensi*	Putative AA sequence	*An. funestus*	Putative AA sequence	*An. plumbeus*	Putative AA sequence
GSTE1 -1	64	224	71	222	62	223	NP	NP
GSTE1-2	78		64		83			
GSTE2-1	74	221	59	221	72	221	64	221
GSTE2-2	90		75		71		77	
GSTE2b-1	NP	NP	NP	NP	NP	NP	68	221
GSTE2b-2	NP		NP		NP	NP	70	
GSTE4	65	225	73	224	65	224	72	64
GSTE5	72	230	66	225	66	224	60	77
GSTE6	91	227	71	222	?	?	?	68
GSTE7-1	76	225	75	223	64	223	105	223
GSTE7-2	66		66		61		71	

NP  =  not present.

?  =  sequence is not known.

GSTEs sequences from *An. funestus*, *An. plumbeus* and *An. stephensi* showed strong similarity to those of *An. gambiae*. Identity of *Anopheles* GST protein sequences (among paralogs) ranged from 45% (between GSTE1 and GSTE6) to 66% (GSTE1 and GSTE2) in *An. gambiae*, from 45% (between GSTE1 and GSTE6) to 70% (between GSTE1 and GSTE2) in *An. stephensi*, from 52% (between GSTE1 and GSTE5) to 72% (between GSTE1 and GSTE2) in *An. funestus* and from 47% (between GSTE2 and GSTE5) to 81% (between GSTE2 and GSTE2B) in *An. plumbeus* (Supplementary Table S4). *GSTE2* was the most conserved gene with no exonic indels observed in the four *Anopheles* species. Comparison among *GSTE2* orthologs showed sequence identity varying from 76.4% to 90.4% (Supplementary Table S4). One indel was found when *Anopheles* GSTE2 sequence were compared to those in *Aedes aegypti* and two when compared to *Drosophila* genes. While codon number was conserved, codon identity was more variable. When compared to the *An. gambiae* GSTE2 sequence 18, 20 and 50 amino acid changes were observed in *An. funestus*, *An. stephensi* and *An. plumbeus*, respectively (Fig. 2). All other genes contained at least one codon indel when compared to *An. gambiae*. For example, three amino acids (positions 92–94) were absent in the second exon of GSTE1 in both *An. stephensi* and *An. funestus* (Fig. 2), at the end of the N-terminal (the G site, where the GSH binds). A similar N-terminus deletion was also found in GSTE4 in *An. funestus*, *An. stephensi* and *An. plumbeus*, when compared to *An. gambiae* (positions 95–96). Fig. 2 (précised in Supplementary Table S5) shows all indels observed in five GSTE genes in *An. stephensi*, *An. funestus* and *An. plumbeus*, when compared to *An. gambiae*.

The length of the intergenic regions were highly variable (Fig. 1) and sequence identity very low, ranging from 17% to 27.6%. Conserved residues found in these regions using MEME tools are shown in Supplementary Table S11.

**Figure 4:**
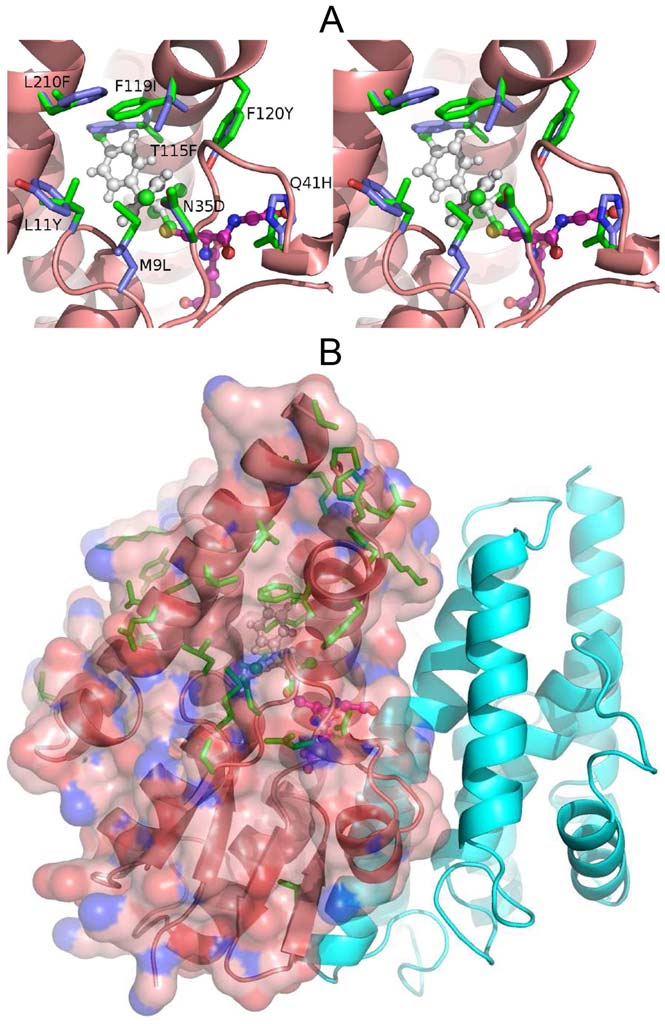
Sequence differences between ApGSTE2 and ApGSTE2B mapped onto structural models of each. A) Overall distribution of differences. All differences are shown with respect to the side chains present in ApGSTE2 on its structural model, represented as both cartoon and surface. Ball and stick representation is used for ligands (white carbon for DDT, as modelled by Wang et al., 2008 [29] into AgGSTE2, magenta for GSH present in crystal structures of AgGSTE2). The position of the second chain of the dimer is shown as a cyan cartoon. B) Cross-eyed stereo close-up of the catalytic site showing nearby sequence differences as sticks (green for ApGSTE2, purple for ApGSTE2B) and ligands as in A). Sequence differences are labelled, for example, as Q41H to indicate that Gln41 in ApGSTE2 is replaced by His in ApGSTE2B.

Molecular models of ApGSTE2 and ApGSTE2B were constructed in order to map sequence differences and predict their potential consequences for activity (Fig. 4). Although differences are found throughout the structure (Fig. 4A), interesting trends are evident. No differences at all are found at the dimer interface and only a single difference (Gln41 in ApGSTE2 vs His in ApGSTE2B) at the glutathione binding site, a difference that allows for conservation of a hydrogen-bonding function. Dimerisation is considered important for catalytic activity [52] so that these two observations together suggest that both paralogous sequences are catalytically active. In sharp contrast, sequence differences are relatively abundant at the H-site as shown in more detail in Fig. 4B. Some can be considered conservative, such as the replacement of Asn35 in ApGSTE2 with Asp, or Phe120 with Tyr. Phe119 and Leu210 in ApGSTE2 are replaced by Ile and Phe, respectively, substantial changes which, nevertheless, may be compensatory in volume and therefore not necessarily causative of large structural changes at the H-site.

### Positive selection

#### Site tests

To identify putatively positively selected codons we compared the likelihood of the data under models which do and models which do not allow for some codons to be under positive selection. Two data sets were analysed, one with all available sequences (GSTall) and one in which the incomplete sequences of *GSTE6* from *An. plumbeus*, *An. darlingi* and *An. funestus* were excluded (GST noE6 pfd). Likelihood ratios tests (LRT) indicated that models which included a proportion of positively selected sites were not significantly more likely for the GSTE cluster than models without positive selection (table 2). In models which allowed two or three site classes, the majority of sites (>95%) were inferred to be under strong purifying selection, with ω≤0.09.

**Table 2:** Likelihood ratio test of positive selection at sites in the GSTE cluster.

Dataset	GSTall		GST no e6 pfd	
**Model**	**lnL**	**2(lnL(Model1)-lnL(Model2))**	**lnL**	**2(lnL(Model1)-lnL(Model2))**
**M1a (neutral)**	−12442.798904	M1a vs M2a (df = 2)0 (NS)	−11652.591604	M1a vs M2a (df = 2)0 (NS)
**M2a (positive selection)**	−12442.798904		−11652.591604	
**M7 (beta)**	−12236.238202	M7 vs M8 (df = 2)5.92, P = 0.052 (NS)	−11464.558968	M7 vs M8 (df = 2)4.97, P = 0.083 (NS)
**M8 (beta and ω)**	−12233.277059		−11462.072748	
**M8a (beta and ω_s_ = 1)**	−12233.277059	M8 vs M8a (df = 1)0 (NS)	−11462.072748	M8 vs M8a (df = 1)0 (NS)

LnL: Log likelihood of the sequence alignment and tree under a particular model.

df: degrees of freedom, NS: not significant.

#### Branch tests

We then tested the hypothesis that positive selection acted on certain branches in the tree by comparing the likelihood of the alignment and tree between branch models. Initially we compared the likelihood of the data under branch model 0, where all branches have the same ω, to branch model 1, where ω is estimated for each branch in the tree (Supplementary Table S6). This is a test of whether there is heterogeneity in ω across the tree. Model 1 is heavily parameterized but is useful for suggesting which branches are likely to be under positive selection. The LRT was significant (GSTall P≤3.73e−^10^), supporting the hypothesis of heterogeneity of ω between branches. Four to five branches had ω>1 in the GSTall tree, three of which were equivalent branches between the GSTall gene set tree and the GST noE6 pfd tree. One of these was the internal branch leading to *GSTE5* (Fig. 3), and examination of the estimated dN and dS for each branch suggested that this branch has an elevated rate of non-synonymous substitution compared to most other branches (dN 0.11, the fourth highest dN in the tree for GSTall). We selected the *GSTE5* branch for further tests of selection, by comparing the likelihood of the data under models where this branch was allowed a different ω from the rest of the tree, either evolving neutrally (ω_1_ = 1, relaxed model 2) or under positive selection (ω_1_≥1, strict model 2, Supplementary Table S6). The LRT comparing model 0 with strict model 2 support the hypothesis of a higher ω in the *GSTE5* branch than the rest of the tree (GSTall P = 1.21e^−06^). However, the LRT comparing the strict versus relaxed model 2 was not significant (GSTall P = 0.20), meaning that this elevated ω could be the result of relaxed selection on the *GSTE5* branch rather than positive selection. The insignificant result might also indicate that the branch models have insignificant power to detect positive selection at a subset of sites in the *GSTE5* branch. We therefore went on to conduct more powerful branch site tests.

#### Branch site tests

**Figure 5:**
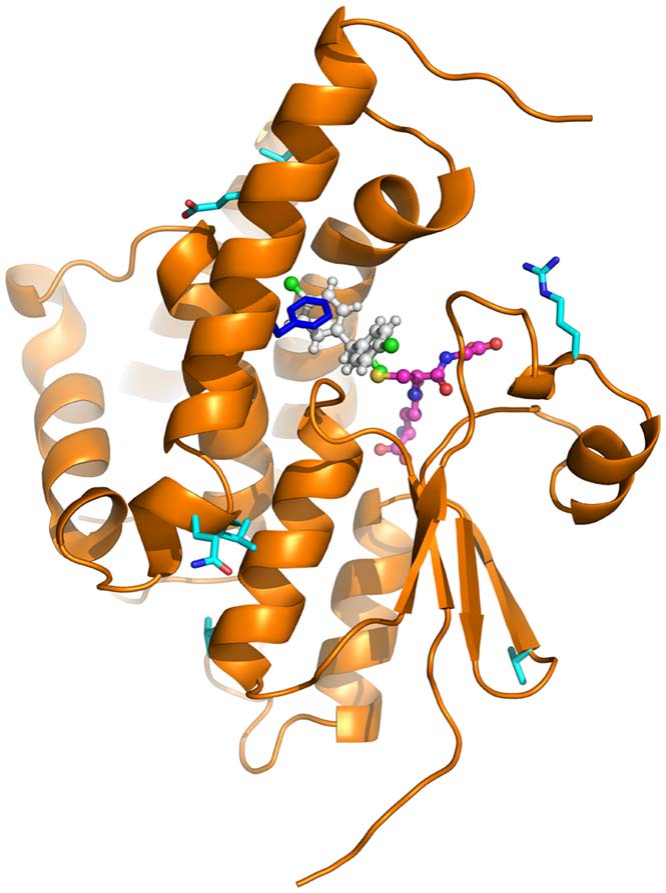
Sites inferred to be under positive selection in GSTE5. Sites under positive selection: ω>1 with P>0.95 in both GSTall and GST no e6 pfd datasets; (see Figure 2) are shown as sticks on a structural model of AgGSTE5. Ball and stick representation is used for ligands (white carbon for DDT, magenta for GSH – see Wang et al. [29]. Phe212, at the heart of the H site is dark blue, other positions cyan.

Model A2, which allows for positive selection at a subset of sites in the foreground branch leading to *GSTE5* (Fig. 3) was favoured in the LRT over model A1, which does not allow for positive selection (GSTall P = 3.05e^−07^, Supplementary Tables S7 and S8). This supports the hypothesis that some sites have been under positive selection in the *GSTE5* lineage. The sites inferred to be under positive selection are shown in figure 2. These sites were mapped onto a structural model of AgGSTE5. One of the positions inferred to be under positive selection Phe212, (position 232 in the alignment shown in figure 2) is located at the heart of the H-site of AgGSTE2, contacting DDT in the binding model predicted by Wang et al. [29] (Fig. 5). None of the other positions is situated near the catalytic site.

### Simulations

The power of the branch site test to detect positive selection at sites on the foreground branch under model A2 at a P≤0.05 for the simulated datasets was reasonable: 70% for ω_2_ = 4, increasing to 98% for ω_2_ = 999 (see Supplementary Table S9). When data were simulated under model A1, the false positive rate (for which positive selection was detected at P≤0.05 although there was none) was 4%. The exact value for ω_2_ inferred by codeml was not accurate at any of the simulated ω_2_ levels, being overestimated for ω_2_ = 4 and ω_2_ = 9, and underestimated for ω_2_ = 999 (data not shown). For the BEB detection of which sites on the foreground branch were under positive selection, the false positive rate was very low (see Supplementary Table S10): a maximum false positive rate of 0.005 (0.5%) was detected for sites with P>0.95 at ω_2_ = 9 and for P>0.99 the false positive rate was extremely low for all simulated ω_2_ values. The accuracy of the BEB procedure, which is the proportion all sites found by codeml to be under positive selection that are really under positive selection was fairly high: the minimum accuracy found was 0.935 for P>0.95 and ω_2_ = 4. However, the power of the BEB procedure for this type of dataset is poor: at best, 0.516 for P>0.95 at ω_2_ = 999 (Supplementary Table S10). Halving the branch lengths with ω_2_ = 9 reduced the power of site and branch tests to detect positive selection from 92% for the real branch lengths to 83% for halved branch lengths, with little effect on the false positive rate. Doubling the branch lengths resulted in a small increase in power to 95%, but a large increase in false positive rate from 4% to 17% (Supplementary Table S9).

### 3′Untranslated regions

3′ RACE PCR sequencing revealed differing 3′ UTR lengths and different locations for the polyadenylation signal among the six different GSTE genes. Three genes (*AsGSTE2*, *ApGSTE2B* and *AfGSTE6*) and the pseudogene (*ψAsGSTE2*) had two different transcripts (Table 3) and *ApGSTE2B* and *AfGSTE6* displayed two polyadenylation signals at different positions. As there were many stop codons, it was not possible to determine the exact size of the *AsGSTE2* 3′UTR. Two different putative poly (A) signals were found in *GSTE6* from *An. funestus* and *GSTE2* from *An. plumbeus*: the most common hexamer in eukaryotes (AAUAAA), and the hexanucleotide AAUAUA, which has been reported previously in Diptera at a lower frequency [53]; [54]. No known polyadenylation signal was found in *AfGSTE5*.

**Table 3:** Location of GSTE polyadenylation signals and 3′ UTR lengths in *An. stephensi*, *An. funestus* and *An. plumbeus*.

Gene	PA site position at the gene after the stop codon andsize of 3′UTR		
	***An. stephensi***	***An. funestus***	***An. plumbeus***
**GSTE1**	+137 (160)	+170 (191)	-
**GSTE2**	+15 (33 or 76)	+40 (64)	+37 or +112 (136)
**GSTE2b**	-	-	+33 and +56 (73 or 96)
**GSTE4**	+84 (109)	+102 (127)	+9 (58)
**GSTE5**	+231 (267)	+? (361)	+38 (62)
**GSTE6**	+332 (352)	+140 or +333 (349 or 416)	?
**GSTE7**	+19 (46)	+23 (45)	+143 (165)

Polyadenylation signal location is numbered relative to final base of stop codon. 3′ UTR lengths are given in parentheses.

While no species-specific motifs were detected using MEME, 10 gene-specific motifs, present in all species, were detected in the 3′UTR. Supplementary Table S11 shows the short sequences obtained by using MEME motif discovery tool.

Within the 3′UTR sequences, twelve potential miRNA targets were identified using miRanda with *An. gambiae* mature miRNAs as input (Supplementary Table S12). Twenty two miRNA targets were identified from comparison of *D. melanogaster* mature miRNAs though 6 of these predictions replicated hits from the *An. gambiae* miRNA search (e.g. dme-mir-9c ≡ aga-mir-9c). For the remaining 10 hits, no *An. gambiae* homologue was identified using MapMi, perhaps indicating that these are false positives. No cross-species conservation of miRNA:mRNA target prediction was noted, however the majority of potential targets were within the 3′UTR of *GSTE5* (16/28 or 57% of all novel hits, or 13/25 or 52% when hits in both *An. stephensi GSTE5a* and *GSTE5b* are counted singly). If the EVD (extreme value distribution) of miRanda scores was computed using sequences 1 kb 3′ of all *An. gambiae* genes (N = 13,621 vs N = 4,033 for true 3′UTRs) then additional miRNA targets were identified (see Supplementary Table S12), however, no cross-species conservation was seen.

### Gene expression

The multiplex assay performed in this study allowed us to compare gene expression across three different life stages: 3^rd^ instar larvae, pupae and adults in *An. gambiae* and *An. stephensi*. In *An. funestus* we had no access to adults and hence RNA was only extracted from larvae and pupae.

With the exception of *AsGSTE6*, results indicate consistent activity for all six target loci included in the analysis during all three life stages in all of the three species (i.e. *An. gambiae, An. stephensi* and *An. funestus*). *AsGSTE6* yielded only detectable products in one of three replicates in the adult stage. Across the life stages gene expression levels fluctuated though most of the loci displayed higher levels during the larval stage with the exception of *GSTE5* in *An. funestus*. Figures S2 and S3 show the results obtained for the multiplex GeXP assays.

## Discussion

In this study the order and sequence of the insect specific GST epsilon (GSTE) cluster of *An. funestus* and *An. stephensi*, both belonging to the *Cellia* subgenus, and of *Anopheles plumbeus*, from the *Anopheles* subgenus, were characterised and compared to those of *An. gambiae*. *An. gambiae* has 8 GSTE genes, the same number as in *A. aegypti* yet only 4 of the 8 genes are recognised orthologues between the two taxa.

*GSTE1* was absent in *An. plumbeus*. *GSTE1* was also not found in a recent transcriptomic analysis of *An. darlingi* (Nyssorhynchus) [55]. This may reflect a secondary loss or a radiation within the lineage leading to the *Cellia* subgenus. Putative orthologs of all other GSTE genes are present in all four *Anopheles* species studied and in the transcriptome of *An. darlingi*, (apart from *ApGSTE2B*, which is *An. plumbeus* specific) indicating that the GSTE expansion predates the (*Cellia-Anopheles*)*-*(*Kerteszia-Lophopodomyia-Nyssorhynchus*) split. As such, we demonstrate that multiple independent duplication events (the duplication leading to *GSTE1* in *Cellia* and the 1−3 duplications necessary to give rise to *GSTE5-7*) must be invoked to explain the pattern of GSTE gene relationships. The consistency in gene order is contrary to our expectations as the rate of rearrangement in gene order between *An. gambiae* and *An. funestus* is the highest reported for eukaryotes [56].

Gene duplication is the major mechanism for generating new genes and the acquisition of novel function [57]. Zhou et al. [58] suggested that it provided the genomic basis for the successful radiation of early eukaryotes. Duplications commonly arise from retrotransposition or unequal crossing over and in the former case the new copy has no intron since it is the result of reverse transcription of an mRNA from a parental gene and usually is inserted in a region distant from the original gene. The GST epsilon class has probably diversified through unequal crossing over resulting in tandem duplication.

Novel duplication may produce different endpoints: 1) the new copy retains the function of the original gene, 2) the new copy accumulates mutations resulting in either functional diversification from the parental gene (neofunctionalization) or adoption of functions previously performed by the parental gene (subfunctionalization), 3) the new copy accumulates deleterious mutations resulting in loss of function, and then either becomes a pseudogene or is lost completely (gene death). In addition to the duplication event that gave rise to *GSTE1* (probably from *GSTE2*), we also have identified a duplicate *GSTE2* in *An. plumbeus*. *GSTE2* has been shown, through QTL mapping, to be associated with resistance of *An. gambiae* to DDT and through biochemical characterisation to be capable of metabolising DDT ([11], [59]). Within the cytochrome P450s, duplication of two P450s in *An. funestus* (*CYP6P3* and *CYP6P9*) has been associated with an insecticide resistance phenotype [60]. It is interesting to speculate as to whether the GSTE2 paralogs in *An. plumbeus* share the same function or have distinctive roles in detoxification of compounds encountered in the tannin-rich environment encountered by this species. Structural modelling suggests that the paralogous sequences are both catalytically active but have different substrate specificities since sequence differences between the two are common at the H site while the glutathione binding site and dimer interface are largely conserved between the two (Fig. 4).

In addition, in this work, we have identified one putative pseudogene, located between *GSTE2* and *GSTE4* in *An. stephensi* (*ψAsGSTE2*). Through sequencing of this region in individuals from both colony material (Beech colony) and field collections from Pakistan and Afghanistan we have demonstrated that this pseudogene is found in all specimens and displays considerable sequence conservation. Through RACE-PCR analysis we demonstrate transcription of *ψAsGSTE2*. Together, these observations suggest some function associated with this ‘pseudogene’. Zheng & Gerstein [61] suggested a classification system for pseudogenes according to their level of functionality. Some pseudogenes are able to regulate gene functions, including that of the parental gene through formation of chimeric mRNA transcripts with those transcribed by neighbouring genes. The record of the pseudogene expression and its high level of conservation among different populations in the present work suggest it is a functional sequence and is deserving of much closer attention. Further studies to characterize the *ψAsGSTE2* mRNA and the predicted protein encoded by the pseudogene and its functional domain are necessary in order to identify a potential role of this sequence in the regulation of other GSTE genes.

Here we show that *GSTE2* displays the highest level of conservation, with no indels in any of the four *Anopheles* species. All other GSTE genes contain at least three indels (see Supplementary Table S5 and Fig. 2). This higher erosion rate may indicate that *GSTE2* plays a pivotal role in *Anopheles* adaptive processes whilst other genes could be more specialized and be more likely to undergo accelerated selection because of their relaxed constraint. As mentioned above AgGSTE2 is the most important GST in conferring DDT resistance in *An. gambiae* and this enzyme displayed the highest DDT dehydrochlorinase activity ever reported for any GST enzyme ([59]; [10]). Its putative ortholog in *Ae. aegypti* is also overexpressed in DDT resistant strains [9].

In order to address the high DDT-detoxifying activity of AgGSTE2, Wang et al. [29] carried out crystallization studies. While the protein structure and glutathione binding mode were successfully elucidated, crystals containing DDT could not be obtained. DDT could, however, be manually positioned in a complementary, V-shaped pocket at the H-site in a suitable orientation for nucleophilic attack by bound glutathione. A somewhat different DDT binding mode has recently been proposed for *D. melanogaster* GSTD1, again based on modelling rather than experimental data, but supported by NMR measurements [62]. However, the evolutionary separation of delta and epsilon class GSTs cautions that they may well bind the same substrate in different fashions. For this reason, we interpreted our data in the light of the binding mode tentatively proposed by Wang et al. [29]. They identify the residues constituting the active site, a pocket in a V-shape, which is responsible for the DDT-binding capability (Leu9, Leu11, Ser12, Pro13, Pro14, Leu36, Leu37, His41, Ile55, Phe108, Met111, Phe115, Leu119, Phe120, Leu207, and Phe210). In addition, the side chains of Arg112, Glu116, and Phe120 form a pocket cap. This cap over the pocket provides a better-sealed hydrophobic pocket increasing DDT affinity, once it is isolated from the outside aqueous environment. Our results show that a change from Thr115 in *ApGSTE2* to Phe in *ApGSTE2B* results in a large, uncompensated change in volume and chemical nature. This difference, with the various smaller substitutions nearby, strongly suggests that the two paralogous sequences are likely to differ in substrate specificity.

Since GSTs play such an important role in the detoxification process of toxic compounds that could be important for adaptation to different habitats, we examined if the GSTE genes show a signature of positive selection. We have demonstrated that at least one gene, *GSTE5*, has in the past evolved under positive selection. Consistently, *GSTE5* harbours the highest number of codon indels (five) suggestive of relaxed selective constraint. We identified several positively selected sites in *GSTE5*, four in the N- terminal domain, where the binding of glutathione occurs (the G-site), and 15 in the H-site, which interacts with substrates. Likewise, comparing 12 related *Drosophila* species, Low et al. [2] identified one gene (*GSTD1*) that was evolving under positive selection, and one specific substitution (glycine → lysine at site 171 in the substrate binding domain) was considered the positively selected site. The selection on *GSTE5* is ancient: it occurred after the *GSTE4/5* gene duplication event but in a common ancestor of the *Cellia* and *Anopheles* subgenera before they split at 90−106 Ma. While the evolution of *GSTE5* may have played a role in adaptation to a new habitat, this signature of positive selection could not have been due to selection by more recent synthetic insecticide exposure.

Overall, the tests conducted on simulated data sets suggest that for the GST dataset, the power of branch site tests to detect positive selection was fairly high, so the inference of positive selection is unlikely to be a false positive. The poor estimation of the exact value of ω_2_ for simulated data sets suggests that the estimation of ω_2_ = 999 for the real dataset may well be inaccurate, but the power and accuracy of the branch site tests means most likely ω_2_ >1. Most of the sites on GSTE5 detected to be under positive selection for the real dataset are likely to be truly under positive selection, but it is likely that many positively selected sites have been missed due to the low power of the BEB detection. Simulations with double and half the true branch lengths imply that the GSTE levels of sequence divergence and saturation of substitutions were in a range favorable to the power and accuracy of site and branch tests and BEB detection of sites under positive selection.

Previous work has shown that individual members of epsilon class GSTs are differentially regulated in *An. gambiae;* five out of eight GSTs are over expressed in a resistant (ZAN/U) compared to a susceptible (KISUMU) strain [7]. Here, we compared gene expression profiles for each of the six GST genes evaluated across the different developmental stages, aiming to provide insight into their functional diversification. Apart from *AsGSE*6, all the genes were expressed across all life stages though the expression levels varied considerably. In general, the GSTs showed elevated expression levels in the L3 stage. This is in accordance with the observation of Huang et al. [63] who found that five GST genes (including two members of the epsilon class) are also over expressed in the larval than in other stages in *Spodoptera litura.* In *Drosophila melanogaster*, from the 10 epsilon members only GSTE1 is highly expressed in all life stages [64]. Other studies have shown the role of upregulated *GSTEs* in stress response and it has been suggested to be a potential biomarker for xenobiotic exposure ([65], [66]).

This is the first work to characterise the 3′UTRs of GSTE members in closely related species. RACE PCR data showed that alternative transcripts are being produced by some GSTE genes, including the pseudogene in *An. stephensi*. Some of these sequences contain more than one polyadenylation site (PA) and some did not present any PA. It is known that longer 3′ UTRs might upregulate genes at the translational level and even direct localization of specific mRNA isoforms [67].

Regulatory elements are short sequences that are involved in the control of gene expression and are often 5 to 20 bp long. Consequently, identifying these regions at a genomic scale is a hard task. However, comparing closely related species aids in the identification of conserved domains. In this work, we have identified a *GSTE2* specific motif, which was present in all four *Anopheles* species. Such motifs and the different GSTE mRNAs described above, which might have different functions, could reflect a complex mechanism of gene regulation in supergene families, playing an important role in divergence in expression that lead to GSTs functional diversification and thus should be further investigated.

We also computationally predicted miRNA target sites in the 3′UTRs of GSTE genes; our results showed that there was no conservation of miRNA target sites across species and GSTE members. We found twelve potential miRNA targets and most of them were within the 3′UTR of *GSTE5*. It is recognized that after gene duplication the expression pattern among newly and parental genes rapidly diverge, which could lead to neofunctionalization. Recently, Li et al. [68] demonstrated that miRNAs are very important in evolving the regulatory patterns of duplicated genes (at least in mammals). However, we cannot conclude that miRNAs regulate GST transcript levels, since the non-conservation of targets could also indicate that these are false positives.

This study provides a set of information from closely related species that aids the understanding of GST superfamily evolution and functional divergence. Studying the structure and function of GSTs is of practical interest and many studies have shown the potential use of GSTs for developing vaccines against worms ([69]; [70]) and other parasites ([71]; [72] and [73]), detection of insecticide residues in DDT-sprayed surfaces [74] and for eliminating environmental toxic compounds [75]. Since resistance to chemical insecticide poses a serious threat to vector control programmes, there is a growing interest among researchers in exploring new insecticides or alternative ways of controlling mosquitoes. Therefore, GSTs, which are the main phase II detoxifying enzymes, should receive appropriate attention, since they are implicated in insecticide metabolism. Knock-down of specific GST members through RNAi is currently underway in our laboratory and might deepen our knowledge about GSTs role in mosquito diversification, as well the mechanisms underlying insecticide resistance.

## Supporting Information

Figure S1 Alignment of DNA sequences containing a putative pseudogene found in *Anopheles stephensi* from different localities. Exonic region shared between *AsGSTE2* and *ψAsGSTE2* is boxed.
(PDF)

Figure S2 Multiplex PCR for *An. gambiae* showing relative expression of six epsilon class GSTs in L3 stage larvae, pupae and adult females.
(PDF)

Figure S3 Multiplex PCR for *An. stephensi* and *An. funestus* showing relative expression of epsilon class GSTs in (A) *An. stephensi* L3 stage larvae, pupae and adult females and (B) *An. funestus* L3 stage larvae and pupae.
(PDF)

Table S1 Accession number of epsilon GSTs.
(DOCX)

Table S2 Site classes under branch site models.
(DOC)

Table S3 Primer sequences used for multiplex PCR (GeXP), qRT-PCR and RACE PCR in *An. gambiae*, *An. stephensi*, *An. funestus* and *An. plumbeus*.
(DOCX)

Table S4 Identity matrix of epsilon class GSTs protein from *An. gambiae*, *An. stephensi*, *An. funestus* and *An. plumbeus*. Numbers in bold are the identities calculated for orthologous genes. Protein names are abbreviated (For instance, G1 = Ag GSTE1). ID = identity of 1.000.
(DOCX)

Table S5 Indels and stop codons in the putative amino acid GSTE sequences of *An. funestus*, *An. stephensi* and *An. plumbeus*. Hyphens indicate deletion. NK: not known. NP: gene is not present and AA: amino acid.
(DOC)

Table S6 Test of positive selection on branches in the GSTE gene tree: comparison of likelihoods under different branch models.
(DOC)

Table S7 Test of positive selection at sites in the GSTe5 branch: comparison of likelihoods of branch site models.
(DOC)

Table S8 Estimated ω values for site classes under branch site models.
(DOC)

Table S9 Power and false positive rate of site and branch tests for simulated datasets.
(DOC)

Table S10 Power, accuracy and false positive rate of the BEB method for detecting sites under positive selection in simulated datasets.
(DOC)

Table S11 Motifs discovered by MEME on the intergenic regions (IR) and 3′UTRs data set.
(DOC)

Table S12 Predicted targets of *An. gambiae* miRNAs (aga-mir-X) or *D. melanogaster* mirRNAs (dme-mir-X) in experimentally determined 3′ UTRs of *An. stephensi*, *An. plumbeus* and *An. funestus* GST genes and sequences 3′ of *An. gambiae* genes (maximum length 1 kb). Potential miRNA targets were identified using miRanda [48], [49].
(DOC)
