# Correction: Comparative Genomics of the Anopheline Glutathione S-Transferase Epsilon Cluster

**DOI:** 10.1371/annotation/1dbf46fc-3f3f-47d0-9605-514bda135ba4

**Published:** 2012-01-27

**Authors:** Constância F.J. Ayres, Pie Müller, Naomi Dyer, Craig S. Wilding, Daniel J. Rigden, Martin J. Donnelly

The middle initials were omitted from the first, fourth, fifth, and sixth authors' names. The correct names are: Constância FJ Ayres, Craig S Wilding, Daniel J Rigden and Martin J Donnelly. The correct citation is: Ayres CFJ, Müller P, Dyer N, Wilding CS, Rigden DJ, et al. (2011) Comparative Genomics of the Anopheline Glutathione S-Transferase Epsilon Cluster. PLoS ONE 6(12): e29237. doi:10.1371/journal.pone.0029237

