# Correction: Comparative Genomics of the Anopheline Glutathione S-Transferase Epsilon Cluster

**DOI:** 10.1371/annotation/02a173a2-0473-49b9-a18e-6e530508640f

**Published:** 2012-01-27

**Authors:** Constância Ayres, Pie Müller, Naomi Dyer, Craig Wilding, Daniel Rigden, Martin Donnelly

There was an error in the Author Contributions. CFJA, PM, ND, CSW, DJR and MJD wrote the paper. 

