# Supplementary material for: Comparative Genomics of the Anopheline Glutathione S-Transferase Epsilon Cluster
Source: PLoS One. 2011 Dec 19;6(12):e29237. doi: 10.1371/journal.pone.0029237 (PMC3242777; doi:10.1371/journal.pone.0029237)
Supplement: Figure S1 — Alignment of DNA sequences containing a putative pseudogene found in Anopheles stephensi from different localities. Exonic region shared between AsGSTE2 and ψAsGSTE2 is boxed. (PDF) [file pone.0029237.s001.pdf]

```
                       10        20        30        40        50        60        70        80        90       100       110       120
Afghanistan   TAACTTGAAGCAGCTTCCTATTGGAGTTATCTTCTCGATTAATCATCAGTTGGCTTTCTTCACATTTGTACTTGTGTGTTTCGTACTTGGTACTGGATGCGAACTGAGTTGTACTTGCTG 120
Pakistan 1    TAACTTGAAGCAGCTTCCTATTGAAGTTATCTTCTCGATTAATCATCAGTTGGCTTTCTTCACATTTGTACTTGTGTGTTTCGTACTTGGTACTGGATGCGAACTGAGCTGTACTTGCTG 120
Pakistan 2    TAACTTGAAGCAGCTTCCTATTGAAGTTATCTTCTCGATTAATCGTCAGTTGGCTTTCTTCACATTTGTACTTGTGTGTTTCGTACTTGGTACTGGATGCGAACTGAGTTGTACTTGCTG 120
Beech colony  TAACTTGAAGCAGCTTCCTATTGAAGTTATCTTCTCGATTAATCATCAGTTGGCTTTCTTCACATTTGTACTTGTGTGTTTCGTACTTGGTACTGGATGCGAACTGAGTTGTACTTGCTG 120

                      130       140       150       160       170       180       190       200       210       220       230       240
Afghanistan   CGGGGCAGAAATTGGGACCATCATTAGCGACCGTCAGGCCATCACAGC-----------------------TTGGAGCTGGTCAACTCTGGCGTGTCTTTCCAACGCAACGATTGCTTCA 217
Pakistan 1    CGGGGCAGAAATTGGGACCATCATTAGCGACCGGCAGACCATCACAGGGCAGTTGAAGGAATCTAATGGTGTTGGAGCTGGTCAGCTTTGGCGTGTCTTTCCAACGCAACGATTGCTTCA 240
Pakistan 2    CGGGGCAGAAATTGGGACCATCATTAGCGACCGGCAGACCATCACAGGGCAGTTGAAGGAATCTAATGGTGTTGGAGCTGGTCAGCTTTGGCGTGTCTTTCCAACGCAACGATTGCTTCA 240
Beech colony  CGGGGCAGAAATTGGGACCATCATTAGCGACCGGCAGACCATCACAGGGCAGTTGAAGGAATCTAATGGTGTTGGAGCTGGTCAGCCTTGGCGTGTCTTTCCAACGCAACGATTGCTTCA 240

                      250       260       270       280       290       300       310       320       330       340       350       360
Afghanistan   AAATGTCTAACTTGGTTCTGTACACGCTTGAACCCACAGTGACACGCTGTAGAGTTAACAGGAAACATACCTGTTTGCAGGTGGTCATATGAAGCCGGAGTTCCTGCAGCTATAGGTCCT 337
Pakistan 1    AAATGTATAAGTTAGTTCTGTACACGCTTGAACCCACTGTGAAACGCTGTAGAGTTAACAGGAAACAGACCTGTTTGCAGGTGGTCATATGAAGCCGGAGTTCCTGCAGGTATAGGTCCT 360
Pakistan 2    AAATGTATAAGTTAGTTCTGTACACGCTTGAACCCACTGTGAAACGCTGTAGAGTTAACAGGAAACAGACCTGTTTGCAGGTGGTCATATGAAGCCGGAGTTCCTGCAGGTATAGGTCCT 360
Beech colony  AAATGTATAAGTTAGTTCTGTACACGCTTGAACCCACTGTGAAACGCTGTAGAGTTAACAGGAAACAGACCTGTTTGCAGGTGGTCATATGAAGCCGGAGTTCCTGCAGGTATAGGTCCT 360

                      370       380       390       400       410       420       430       440       450       460       470       480
Afghanistan   TGCCGATGTCCATTATGGTGGTATTTTCTATTGAGCGATCGTTATAGCTTAACCCTCAACATGCGAACCTGGTGCTGGATGATGATGGTACGATCATCACCGAGAGTCATGCGATCATGA 457
Pakistan 1    TGTCGATATCCATTATGGTGGTATTTTCTATTGGGCGATCGTTACAGCTTAACCCTCAACATGCGATCCTGGTGCTGGATGATGAAGGTACGATCATCACCGAGAGTCATGCGATC---- 476
Pakistan 2    TGTCGATATCCATTATGGTGGTATTTTCTATTGGGCGATCGTTACAGCTTAACCCTCAACATGCGATCCTGGTGCTGGATGATGAAGGTACGATCATCACCGAGAGTCATGCGATC---- 476
Beech colony  TGTCGATATCCATTATGGTGGTATTTTCTATTGGGCGATCGTTACAGCTTAACCCTCAACATGCGATCCTGGTGCTGGATGATGAAGGTACGATCATCACCGAGAGTCATGCGATC---- 476

                      490       500       510       520       530       540       550       560       570       580       590       600
Afghanistan   TCTATCTGGTGCCAGTCTTAACACGACAGGACCGGGGTTCAAATCGCATCTAAGCCGTTCCCCCCATAGTGAGGCCAAGACTTCTCGAGGTTGTA------AGGAAGAAGAAGAAGGAAC 571
Pakistan 1    --TATCTGGTGTCAGTCTTAACACGGCAGGACCGGGGTTAAAATCGCATCCAAGCCGTTCCCCCCATAGTGAGGCCAAGACTTCTCGAGGTTGTAGTGCCAAGGAAGAAGAAGAAGGAAC 594
Pakistan 2    --TATCTGGTGTCAGTCTTAACACGGCAGGACCGGGGTTAAAATCGCATCCAAGCCGTTCCCCCCATAGTGAGGCCAAGACTTCTCGAGGTTGTAGTGCCAAGGAAGAAGAAGAAGGAAC 594
Beech colony  --TATCTGGTGTCAGTCTTAACACGGCAGGACCGGGGTTAAAATCGCATCCAAGCCGTTCCCCCCATAGTGAGGCCAAGACTTCTCGAGGTTGTAGTGCCAAGGAAGAAGAAGAAGGAAC 594

                      610       620       630       640       650       660       670       680       690       700       710       720
Afghanistan   CGATTGAGGGAAG--TTCACATTTGCCAAGAAGGAAGAAAATGGTGAAGTTTTTAACTCAAATGCGATTTTTATGACCGAAAACACCAGTACATCAAGAGTATAACAGAAACAGTTAAAT 689
Pakistan 1    CGATTTAGGGAAGTGTTCATATTTGCCAAGAAGAGAGAAAATGGTGAAGCTTTTAACTCAAAAGCGATTTTTATGACCGAAAAC--CAGTACATCAAGAGTAAAACAGAAACAGTTAAAT 712
Pakistan 2    CGATTTAGGGAAGTGTTCATATTTGCCAAGAAGAAAGAAAATGGTGAAGCTTTTAACTCAAAAGCGATTTTTATGACCGAAAAC--CAGTACATCAAGAGTAAAACAGAAACAGTTAAAT 712
Beech colony  CGATTTAGGGAAGTGTTCATATTTGCCAAGAAGAAAGAAAATGGTGAAGCTTTTAACTCAAAAGCGATTTTTATGACCGAAAAC--CAGTACATCAAGAGTAAAACAGAAACAGTTAAAT 712

                      730       740       750       760       770       780       790       800       810       820       830       840
Afghanistan   CTTATCATGCACTGATAGCTAGACAATAAAACGAGAAGTAAGTGGCTATTAAAAAAGAAAATTTGATACAAGTCGGAATCAAGGTGCATGTAAAGAAACGCTAAAGAAAGACAAACAAAT 809
Pakistan 1    CTTATCATGCACTGATAGCTAGACAATAAAACGAGAAGTAAGTGGCTATTAAAAATGAAAATTTGATACAAGTCGGAATCAAGGTGCATGTAAAGAAACGCTAAAGAAAGACAAACAAAT 832
Pakistan 2    CTTATCATGCACTGATAGCTAGACAATAAAACGAGAAGTAAGTGGCTATTAAAAAAGAAAATTTGATACAAGTCGGAATCAAGGTGCATGTAAAGAAACGCGAAAGAAAGACGAACAAAT 832
Beech colony  CTTATCATGCACTGATAGCTAGACAATAAAACGAGAAGTAAGTGGCTATTAAAAAAGAAAATTTGATACAAGTCGGAATCAAGGTGCATGTAAAGAAACGCGAAAGAAAGACGAACAAAT 832

                      850       860       870       880       890       900       910       920       930       940       950       960
Afghanistan   CGTTAAAGTTGACAAAACCAAAAAAGAAGAAAAAAAAAACAGAAGAAAAAGGCAAAGATTACAGTAGTTATCAAGCGCACAGGGCAGAAGTAGCAACAACAGTAGATAATAAAACATTTG 929
Pakistan 1    CGTTAAAGTTGACAAAACCAAAAAAGAAGAAAAAAAAAACAGAAGAAAAAGGCAAAGATTACAGTAGTTATCAAGCGCACAGGACAGAAGTAGCAACAACAGTAGATAATAAAACATTTG 952
Pakistan 2    TGTTAAAGTTGACAAAACCAAAAAAGAAACAAAAAAAAA--GAAGAAAAAGGCAAAGATTACAGTAGTTATCAAGCGCACAGGACAGAAGTAGCAACAACAGTAGATAATAAAACATTTG 950
Beech colony  TGTTAAAGTTGACAAAACCAAAAAAGAAACAAAAAAGAA--GAAGAAAAAGGCAAAGATTACAGTAGTTATCAAGCGCACAGGACAGAAGTAGCAACAACAGTAGATAATAAAACATTTG 950

                      970
Afghanistan   TTCGCTTGCCGGCAT 944
Pakistan 1    TTCGCTTGCCGGCAT 967
Pakistan 2    TTCGCTTGCCGGCAT 965
Beech colony  TTCGCTTGCCGGCAT 965
```
