# Supplementary figures and images for: Comparative Genomics of the Anopheline Glutathione S-Transferase Epsilon Cluster
Source: PLoS One. 2011 Dec 19;6(12):e29237. doi: 10.1371/journal.pone.0029237 (PMC3242777; doi:10.1371/journal.pone.0029237)

# *An. gambiae* Multiplex PCR results

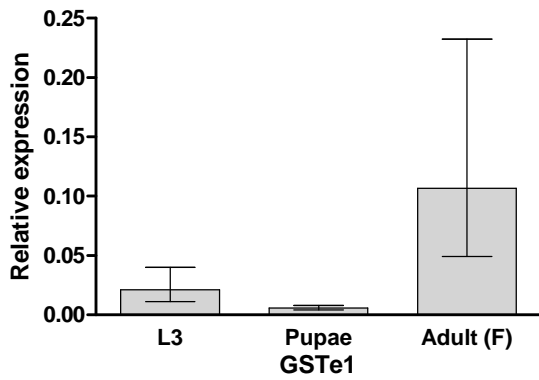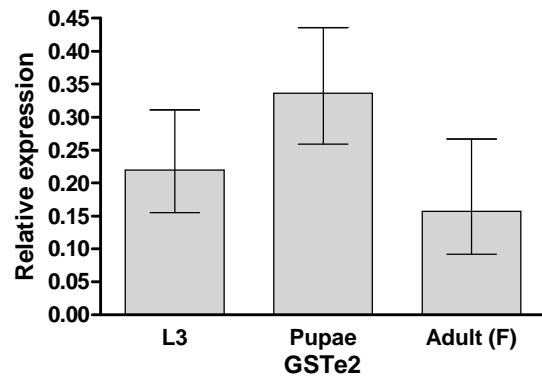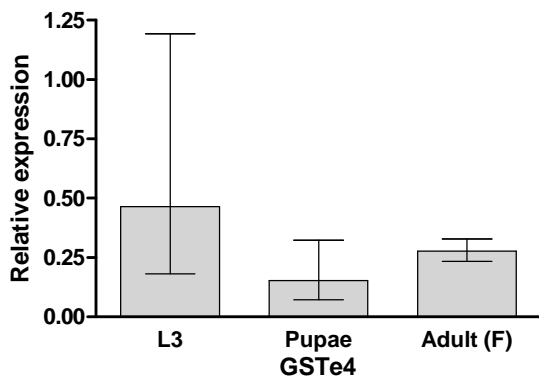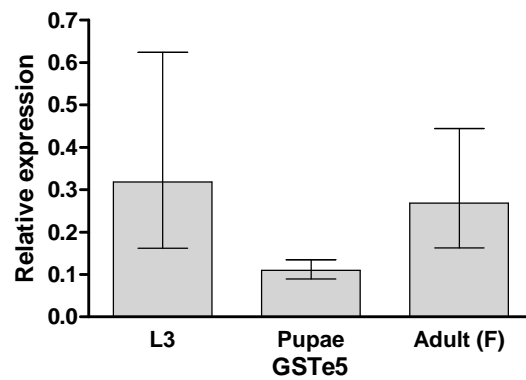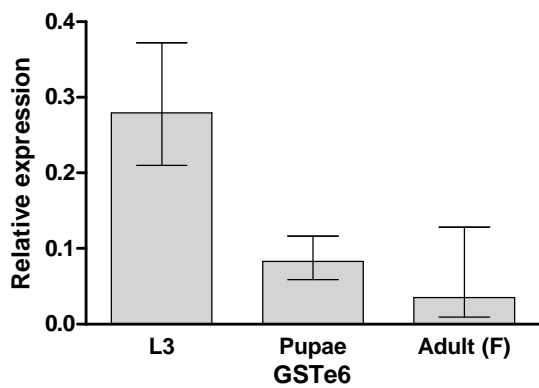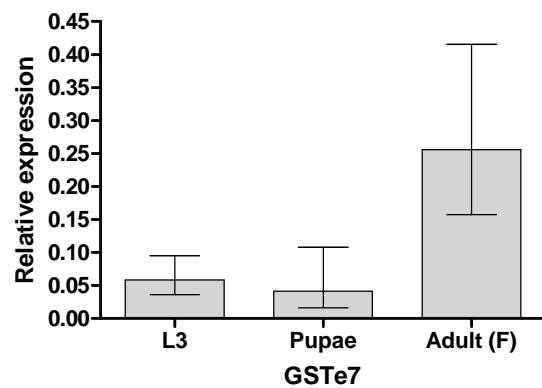

Supplement: Figure S2 — Multiplex PCR for An. gambiae showing relative expression of six epsilon class GSTs in L3 stage larvae, pupae and adult females. (PDF) [file pone.0029237.s002.pdf]

# An. stephensi and An. funestus Multiplex PCR results

A

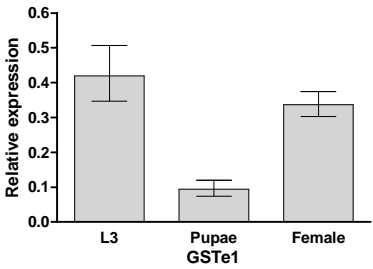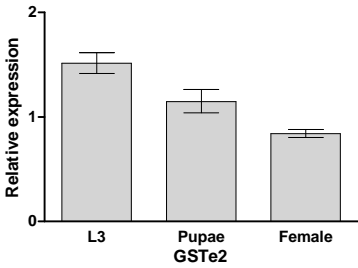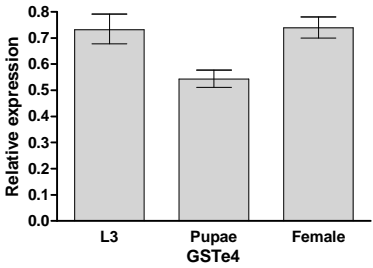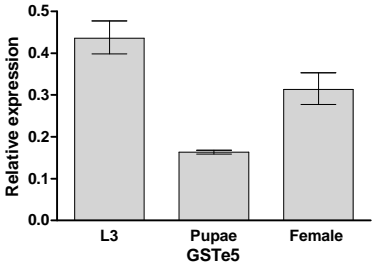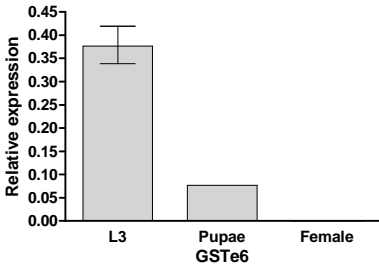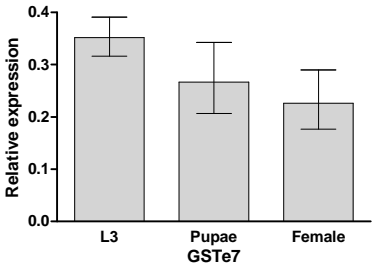

B

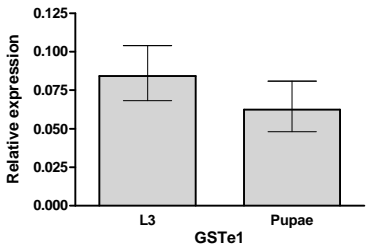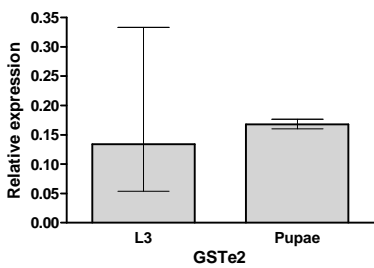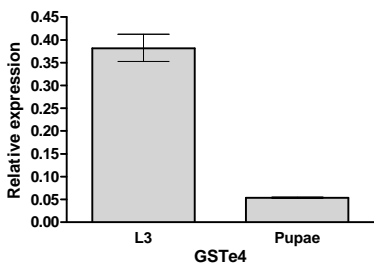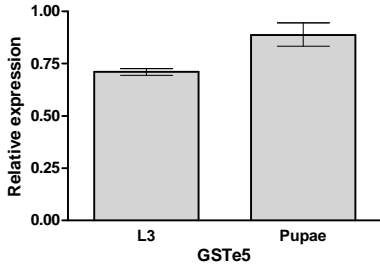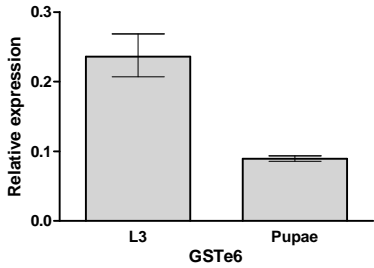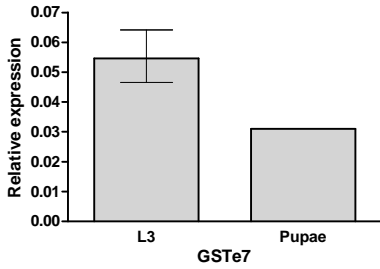

Supplement: Figure S3 — Multiplex PCR for An. stephensi and An. funestus showing relative expression of epsilon class GSTs in (A) An. stephensi L3 stage larvae, pupae and adult females and (B) An. funestus L3 stage larvae and pupae. (PDF) [file pone.0029237.s003.pdf]
