# Supplementary material for: Comparative Genomics of the Anopheline Glutathione S-Transferase Epsilon Cluster
Source: PLoS One. 2011 Dec 19;6(12):e29237. doi: 10.1371/journal.pone.0029237 (PMC3242777; doi:10.1371/journal.pone.0029237)
Supplement: Table S1 — Accession number of epsilon GSTs. (DOCX) [file pone.0029237.s004.docx]

Supplementary Table S1: Accession number of epsilon GSTs

| **Gene** | **GenBank accession numbers** |
| --- | --- |
| AsGSTE1 | HQ418396 |
| AfGSTE1 | HQ418402 |
| AsGSTE2 | HQ418397 |
| AfGSTE2 | HQ418403 |
| ApGSTE2 | HQ418408 |
| ApGSTE2b | HQ418409 |
| AsGSTE4 | HQ418398 |
| AfGSTE4 | HQ418404 |
| ApGSTE4 | HQ418410 |
| AsGSTE5 | HQ418399 |
| AfGSTE5 | HQ418405 |
| ApGSTE5 | HQ418411 |
| AsGSTE6 | HQ418400 |
| AfGSTE6 | HQ418406 |
| ApGSTE6 | HQ418412 |
| AsGSTE7 | HQ418401 |
| AfGSTE7 | HQ418407 |
| ApGSTE7 | HQ418413 |
| ΨAsGSTE2 | JQ031268 |
