# Supplementary material for: Comparative Genomics of the Anopheline Glutathione S-Transferase Epsilon Cluster
Source: PLoS One. 2011 Dec 19;6(12):e29237. doi: 10.1371/journal.pone.0029237 (PMC3242777; doi:10.1371/journal.pone.0029237)
Supplement: Table S2 — Site classes under branch site models. (DOC) [file pone.0029237.s005.doc]

Supplementary Table S2: Site classes under branch site models

| Site Class | Branch site model A with ω_2_ fixed (null) A_1_ | | Modified branch site model A (positive selection) A_2_ | |
| --- | --- | --- | --- | --- |
| Background | Foreground | Background | Foreground |
| 0 | 0<ω_0_<1 | 0<ω_0_<1 | 0<ω_0_<1 | 0<ω_0_<1 |
| 1 | ω_1_=1 | ω_1_=1 | ω_1_=1 | ω_1_=1 |
| 2a | 0<ω_0_<1 | ω_2_=1 | 0<ω_0_<1 | ω_2_>1 |
| 2b | ω_1_=1 | ω_2_=1 | ω_1_=1 | ω_2_>1 |
