# Supplementary material for: Comparative Genomics of the Anopheline Glutathione S-Transferase Epsilon Cluster
Source: PLoS One. 2011 Dec 19;6(12):e29237. doi: 10.1371/journal.pone.0029237 (PMC3242777; doi:10.1371/journal.pone.0029237)
Supplement: Table S3 — Primer sequences used for multiplex PCR (GeXP), qRT-PCR and RACE PCR in An. gambiae, An. stephensi, An. funestus and An. plumbeus. (DOCX) [file pone.0029237.s006.docx]

Supplementary Table S3: Primer sequences used for multiplex PCR (GeXP), qRT-PCR and RACE PCR in *An. gambiae*, *An. stephensi*, *An. funestus* and *An. plumbeus*.

| (GeXP) in *Anopheles stephensi* | | | | | | |
| --- | --- | --- | --- | --- | --- | --- |
| **Gene** | **Primer Forward** | | **Primer Reverse** | | | **cDNA amplicon (bp)** |
| GSTE1 | CGTGTCGAGCAGTGGAACTA | | TGACTTCAAGAATTCCGGCT | | | 107 |
| GSTE2 | ATCGCGTTGAGTATGTGCAG | | CAGCTAAAGTCGGCAATCGT | | | 100 |
| GSTE4 | GTACGGTTCCGACGAGAGTC | | TTCCAAATAGAACCGCAACC | | | 115 |
| GSTE5 | ACATGCAGAAGGGCTACGAG | | CTACGATCCATCGGGAAAAA | | | 136 |
| GSTE6 | TTCTGCACGGACAATCTGAC | | TGCTGACACAGCTCAAGTCC | | | 157 |
| GSTE7 | AACCGGTAATTTATTCGGGC | | GAGATGCAGCTAAAATCGGC | | | 145 |
| SP7 | CATTTCGTTGTGAACCCAAA | | AGTTCATCTCCAGCTCCAGG | | | 128 |
| (GeXP) in *Anopheles gambiae* | | | | | | |
| GSTE1 | GTATTTTTCGCACGCAAACC | | ATAGAGGCGACCGAGGAAAT | | | 155 |
| GSTE2 | CTGCGAAAATGTCCAACCTT | | TTTGCCATACTTCGTCACCA | | | 251 |
| GSTE4 | CGCCATCAATGTGTATCTGG | | CGGTCGCTCCGTAGTACAGT | | | 173 |
| GSTE5 | GAACCCCATCATCAAGCTGT | | TTGGGACGATGTCTAGGGAC | | | 101 |
| GSTE6 | GGCCCTCTTCTTCGAGAGTT | | GCACGCTGTAGGTTCTCCTC | | | 108 |
| GSTE7 | GCTGGACGAGTCCAAGTTCC | | GCATTGGCTTCCTTCTTGC | | | 135 |
| (GeXP) in *Anopheles funestus* | | | | | | |
| GSTE1 | TTGCCTCTACGGTTGGTTTT | | CGATGCCATTGAGTTCTTCA | | | 111 |
| GSTE2 | GGGATTGGAACTGGAACAGA | | TTTGCCATACTTCGTCACCA | | | 169 |
| GSTE4 | TATTGTTGGCAATCGGATGA | | TGTAGTATGGCAACTTGGCG | | | 143 |
| GSTE5 | TAGCTGCATCGCAACGATAG | | AAAGTGTGGCTCAACCGTTC | | | 102 |
| GSTE6 | GAGCGATAGAGGGTTTGCAG | | AAGGTTTTTGGAAAATCGGC | | | 154 |
| GSTE7 | AATCGTTATCACCGCCAGTC | | ATGACTCCCGCTTCAAGATG | | | 135 |
| RACE PCR in *An. stephensi* | | | | | | |
| GSTE1A | | GGATTGGACTTGGAGCGTAGAACC |  | |  | |
| GSTE1B | | TGAAGTCAACCCGAAGCATACGAT |  | |  | |
| GSTE2A | | ACCATTTGCAGCCGGAGTTCTTGA |  | |  | |
| GSTE2B | | CCCGGTGCTGGATGATGAT |  | |  | |
| GSTE4A | | CGGGTCGGGCGGTAGAGTTGA |  | |  | |
| GSTE4B | | GTCACCGAGAGCCACGCAATCA |  | |  | |
| GSTE5A | | CGTCACGCGGGCAAAAGT |  | |  | |
| GSTE5B | | ACTTTGACTCGGGTGTGCTGTTCT |  | |  | |
| GSTE6A | | CACGGACCTCTACACGGACGACTT |  | |  | |
| GSTE6B | | GCGCTTCTGCACGGACAATCT |  | |  | |
| GSTE7A | | GGAGGAGTCGTTATCACCGCCAGTC |  | |  | |
| GSTE7B | | CGCCATCATGATTTATCTCGTGTG |  | |  | |
| PSEUDOA | | GCTTGAACCCACTGTGAAACGCTGTAGAG |  | |  | |
| PSEUDOB | | CCTGGTGCTGGATGATGAAG |  | |  | |
| RACE PCR in *An. funestus* | | | | | | |
| GSTE1A | TATACACGGTCCATCTTAGTCCAC | |  | |  | |
| GSTE1B | CTCTACCCGTCCGATATTGTCCAG | |  | |  | |
| GSTE2A | TTAAACCCCCAACATACGAT | |  | |  | |
| GSTE2B | CCCGGTGCTGGATGATGATGGT | |  | |  | |
| GSTE4A | GAAATCATCCCCATCAATCT | |  | |  | |
| GSTE4B | TTCGGAAGTTAAACCCCCAGCACACG | |  | |  | |
| GSTE5A | TTGACTCCGGTGTGCTGTTCTC | |  | |  | |
| GSTE5B | GCGGTTGCGATTCTATTTC | |  | |  | |
| GSTE6A | GAGCAGCAGGCCCGTATCAATG | |  | |  | |
| GSTE6B | GCTGGGCAAGAGTGAAATCC | |  | |  | |
| GSTE7A | CCGCTCGTGCCCTTGGTAT | |  | |  | |
| GSTE7B | TAGAGTTGATCGAGAAGGAGA | |  | |  | |
| RACE PCR in *An.plumbeus* | | | | | | |
| GSTE2A | AAGGCGTTGGGGCTGGAGTTGG | | |  |  | |
| GSTE2B | TCGGTGAATCTGCTTGCGGGTGAC | | |  |  | |
| GSTE2bA | ATCTTTGAACGCATTATTTACTT | | |  |  | |
| GSTE2bB | CACATGACCATTGCCGATTTTAGC | | |  |  | |
| GSTE4A | AACCCCCAGCACACGATTCC | | |  |  | |
| GSTE4B | AAAATCAACTCGGCCCTCCACTTC | | |  |  | |
| GSTE5A | GCTCGTCGGAAGTGCCTCAG | | |  |  | |
| GSTE5B | GGGATGACGCTGGCCGACCTAAG | | |  |  | |
| GSTE6A | CTAAAAGCGGCCGGCGAGATGATAAAGAT | | |  |  | |
| GSTE6B | AGGGAGGACGGGCGACAACACTGATGGT | | |  |  | |
| GSTE7A | TCGGGCGGTGAAGATGACTGC | | |  |  | |
| GSTE7B | GCACTCGGGCTAGACCTGACC | | |  |  | |
