# Supplementary material for: Comparative Genomics of the Anopheline Glutathione S-Transferase Epsilon Cluster
Source: PLoS One. 2011 Dec 19;6(12):e29237. doi: 10.1371/journal.pone.0029237 (PMC3242777; doi:10.1371/journal.pone.0029237)
Supplement: Table S4 — Identity matrix of epsilon class GSTs protein from An. gambiae, An. stephensi, An. funestus and An. plumbeus. Numbers in bold are the identities calculated for orthologous genes. Protein names are abbreviated (For instance, G1 = Ag GSTE1). ID = identity of 1.000. (DOCX) [file pone.0029237.s007.docx]

Supplementary Table S4: Identity matrix of epsilon class GSTs protein from *An. gambiae*, *An. stephensi*, *An. funestus* and *An. plumbeus*. Numbers in bold are the identities calculated for orthologous genes. Protein names are abbreviated (For instance, G1 = Ag GSTE1). ID = identity of 1.000.

|  | G1 | S1 | F1 | G2 | S2 | F2 | P2 | P2b | G4 | S4 | F4 | P4 | G5 | S5 | F5 | P5 | G6 | S6 | F6 | P6 | G7 | S7 | F7 | P7 |
| --- | --- | --- | --- | --- | --- | --- | --- | --- | --- | --- | --- | --- | --- | --- | --- | --- | --- | --- | --- | --- | --- | --- | --- | --- |
| G1 | ID | **0.804** | **0.787** | 0.660 | 0.674 | 0.687 | 0.691 | 0.669 | 0.526 | 0.515 | 0.515 | 0.495 | 0.497 | 0.517 | 0.508 | 0.488 | 0.448 | 0.473 | 0.377 | 0.364 | 0.548 | 0.530 | 0.566 | 0.557 |
| S1 | 0.804 | ID | **0.834** | 0.693 | 0.702 | 0.711 | 0.707 | 0.698 | 0.511 | 0.513 | 0.513 | 0.484 | 0.471 | 0.500 | 0.486 | 0.493 | 0.447 | 0.451 | 0.354 | 0.353 | 0.575 | 0.553 | 0.571 | 0.580 |
| F1 | 0.787 | 0.834 | ID | 0.704 | 0.708 | 0.721 | 0.708 | 0.713 | 0.526 | 0.533 | 0.533 | 0.517 | 0.512 | 0.519 | 0.519 | 0.526 | 0.445 | 0.444 | 0.352 | 0.346 | 0.582 | 0.551 | 0.578 | 0.578 |
| G2 | 0.660 | 0.693 | 0.704 | ID | **0.904** | **0.918** | **0.773** | **0.855** | 0.533 | 0.553 | 0.553 | 0.555 | 0.493 | 0.539 | 0.539 | 0.497 | 0.406 | 0.440 | 0.342 | 0.324 | 0.582 | 0.578 | 0.596 | 0.623 |
| S2 | 0.674 | 0.702 | 0.708 | 0.904 | ID | **0.923** | **0.764** | **0.837** | 0.546 | 0.557 | 0.557 | 0.546 | 0.497 | 0.539 | 0.517 | 0.488 | 0.397 | 0.426 | 0.328 | 0.311 | 0.573 | 0.582 | 0.596 | 0.600 |
| F2 | 0.687 | 0.711 | 0.721 | 0.918 | 0.923 | ID | **0.778** | **0.864** | 0.528 | 0.539 | 0.530 | 0.533 | 0.497 | 0.522 | 0.513 | 0.488 | 0.414 | 0.448 | 0.342 | 0.324 | 0.573 | 0.569 | 0.587 | 0.609 |
| P2 | 0.691 | 0.707 | 0.708 | 0.773 | 0.764 | 0.778 | ID | **0.814** | 0.511 | 0.548 | 0.535 | 0.546 | 0.476 | 0.491 | 0.495 | 0.475 | 0.427 | 0.453 | 0.346 | 0.328 | 0.560 | 0.542 | 0.569 | 0.573 |
| P2b | 0.669 | 0.698 | 0.713 | 0.855 | 0.837 | 0.864 | 0.814 | ID | 0.506 | 0.539 | 0.539 | 0.533 | 0.493 | 0.513 | 0.517 | 0.497 | 0.423 | 0.453 | 0.346 | 0.337 | 0.591 | 0.578 | 0.596 | 0.605 |
| G4 | 0.526 | 0.511 | 0.526 | 0.533 | 0.546 | 0.528 | 0.511 | 0.506 | ID | **0.792** | **0.792** | **0.777** | 0.606 | 0.641 | 0.637 | 0.600 | 0.364 | 0.373 | 0.300 | 0.293 | 0.454 | 0.456 | 0.456 | 0.434 |
| S4 | 0.515 | 0.513 | 0.533 | 0.553 | 0.557 | 0.539 | 0.548 | 0.539 | 0.792 | ID | **0.857** | **0.803** | 0.614 | 0.668 | 0.659 | 0.617 | 0.387 | 0.401 | 0.306 | 0.317 | 0.460 | 0.462 | 0.480 | 0.462 |
| F4 | 0.515 | 0.513 | 0.533 | 0.553 | 0.557 | 0.530 | 0.535 | 0.539 | 0.792 | 0.857 | ID | **0.776** | 0.597 | 0.637 | 0.641 | 0.604 | 0.362 | 0.388 | 0.320 | 0.321 | 0.451 | 0.449 | 0.453 | 0.436 |
| P4 | 0.495 | 0.484 | 0.517 | 0.555 | 0.546 | 0.533 | 0.546 | 0.533 | 0.777 | 0.803 | 0.776 | ID | 0.595 | 0.644 | 0.640 | 0.607 | 0.385 | 0.390 | 0.299 | 0.309 | 0.458 | 0.446 | 0.464 | 0.446 |
| G5 | 0.497 | 0.471 | 0.512 | 0.493 | 0.497 | 0.497 | 0.476 | 0.493 | 0.606 | 0.614 | 0.597 | 0.595 | ID | **0.839** | **0.847** | **0.756** | 0.374 | 0.383 | 0.308 | 0.280 | 0.442 | 0.435 | 0.439 | 0.413 |
| S5 | 0.517 | 0.500 | 0.519 | 0.539 | 0.539 | 0.522 | 0.491 | 0.513 | 0.641 | 0.668 | 0.637 | 0.644 | 0.839 | ID | **0.915** | **0.777** | 0.370 | 0.375 | 0.302 | 0.290 | 0.486 | 0.480 | 0.480 | 0.449 |
| F5 | 0.508 | 0.486 | 0.519 | 0.539 | 0.517 | 0.513 | 0.495 | 0.517 | 0.637 | 0.659 | 0.641 | 0.640 | 0.847 | 0.915 | ID | **0.781** | 0.387 | 0.397 | 0.315 | 0.290 | 0.473 | 0.471 | 0.471 | 0.449 |
| P5 | 0.488 | 0.493 | 0.526 | 0.497 | 0.488 | 0.488 | 0.475 | 0.497 | 0.600 | 0.617 | 0.604 | 0.607 | 0.756 | 0.777 | 0.781 | ID | 0.379 | 0.371 | 0.315 | 0.299 | 0.462 | 0.442 | 0.455 | 0.433 |
| G6 | 0.448 | 0.447 | 0.445 | 0.406 | 0.397 | 0.414 | 0.427 | 0.423 | 0.364 | 0.387 | 0.362 | 0.385 | 0.374 | 0.370 | 0.387 | 0.379 | ID | **0.859** | **0.625** | **0.524** | 0.385 | 0.384 | 0.384 | 0.366 |
| S6 | 0.473 | 0.451 | 0.444 | 0.440 | 0.426 | 0.448 | 0.453 | 0.453 | 0.373 | 0.401 | 0.388 | 0.390 | 0.383 | 0.375 | 0.397 | 0.371 | 0.859 | ID | **0.671** | **0.557** | 0.366 | 0.374 | 0.387 | 0.383 |
| F6 | 0.377 | 0.354 | 0.352 | 0.342 | 0.328 | 0.342 | 0.346 | 0.346 | 0.300 | 0.306 | 0.320 | 0.299 | 0.308 | 0.302 | 0.315 | 0.315 | 0.625 | 0.671 | ID | **0.661** | 0.296 | 0.312 | 0.316 | 0.308 |
| P6 | 0.364 | 0.353 | 0.346 | 0.324 | 0.311 | 0.324 | 0.328 | 0.337 | 0.293 | 0.317 | 0.321 | 0.309 | 0.280 | 0.290 | 0.290 | 0.299 | 0.524 | 0.557 | 0.661 | ID | 0.295 | 0.302 | 0.311 | 0.306 |
| G7 | 0.548 | 0.575 | 0.582 | 0.582 | 0.573 | 0.573 | 0.560 | 0.591 | 0.454 | 0.460 | 0.451 | 0.458 | 0.442 | 0.486 | 0.473 | 0.462 | 0.385 | 0.366 | 0.296 | 0.295 | ID | **0.888** | **0.893** | **0.786** |
| S7 | 0.530 | 0.553 | 0.551 | 0.578 | 0.582 | 0.569 | 0.542 | 0.578 | 0.456 | 0.462 | 0.449 | 0.446 | 0.435 | 0.480 | 0.471 | 0.442 | 0.384 | 0.374 | 0.312 | 0.302 | 0.888 | ID | **0.901** | **0.802** |
| F7 | 0.566 | 0.571 | 0.578 | 0.596 | 0.596 | 0.587 | 0.569 | 0.596 | 0.456 | 0.480 | 0.453 | 0.464 | 0.439 | 0.480 | 0.471 | 0.455 | 0.384 | 0.387 | 0.316 | 0.311 | 0.893 | 0.901 | ID | **0.811** |
| P7 | 0.557 | 0.580 | 0.578 | 0.623 | 0.600 | 0.609 | 0.573 | 0.605 | 0.434 | 0.462 | 0.436 | 0.446 | 0.413 | 0.449 | 0.449 | 0.433 | 0.366 | 0.383 | 0.308 | 0.306 | 0.786 | 0.802 | 0.811 | ID |
