# Supplementary material for: Comparative Genomics of the Anopheline Glutathione S-Transferase Epsilon Cluster
Source: PLoS One. 2011 Dec 19;6(12):e29237. doi: 10.1371/journal.pone.0029237 (PMC3242777; doi:10.1371/journal.pone.0029237)
Supplement: Table S5 — Indels and stop codons in the putative amino acid GSTE sequences of An. funestus, An. stephensi and An. plumbeus. Hyphens indicate deletion. NK: not known. NP: gene is not present and AA: amino acid. (DOC) [file pone.0029237.s008.doc]

Supplementary Table S5: Indels and stop codons in the putative amino acid GSTE sequences of *An. funestus*, *An. stephensi* and *An. plumbeus*. Hyphens indicate deletion. NK: not known. NP: gene is not present and AA: amino acid.

| **Gene** | **AA position** | ***An. gambiae*** | ***An. funestus*** | ***An. stephensi*** | ***An. plumbeus*** |
| --- | --- | --- | --- | --- | --- |
| GSTE1 | 82 | Gly | - | - | NP |
|  | 83 | Glu | - | - |  |
|  | 84 | Gly | - | - |  |
|  | 195 | - | - | Lys |  |
|  | 223 | - | Thr | - |  |
|  | 224 | - | Lys | - |  |
|  | stop | TAA | TGA | TAA |  |
| GSTE2 | stop | TAA | TAA | TAG | TAA |
| GSTE2B | stop | NP | NP | NP | TAA |
| GSTE4 | 4 | - | Ala | Lys | - |
|  | 83 | Pro | - | - | - |
|  | 84 | Glu | - | - | - |
|  | stop | TGA | TGA | TGA | TAA |
| GSTE5 | 3 | Thr | - | - | - |
|  | 5 | Pro | - | - | - |
|  | 6 | Ile | - | - | - |
|  | 84 | Lys | - | Arg | - |
|  | 224 | Gly | - | - | - |
|  | 226 | Ser | - | - | - |
|  | 227 | Val | Ile | Ile | - |
|  | 228 | Ala | Lys | Lys | - |
|  | stop | TAA | TAA | TAA | TAA |
| GSTE7 | 2 | Glu | Asp | Glu | - |
|  | 3 | Pro | - | - | - |
|  | 4 | Ser | - | - | Ser |
|  | stop | TGA | TGA | TAA | TGA |
