# Supplementary material for: Comparative Genomics of the Anopheline Glutathione S-Transferase Epsilon Cluster
Source: PLoS One. 2011 Dec 19;6(12):e29237. doi: 10.1371/journal.pone.0029237 (PMC3242777; doi:10.1371/journal.pone.0029237)
Supplement: Table S6 — Test of positive selection on branches in the GSTE gene tree: comparison of likelihoods under different branch models. (DOC) [file pone.0029237.s009.doc]

Supplementary Table S6: Test of positive selection on branches in the GSTE gene tree: comparison of likelihoods under different branch models

| **Dataset** | **GSTall** | | | **GST no e6pfd** | | |
| --- | --- | --- | --- | --- | --- | --- |
| **Model** | **lnL** | **Parameters estimated** | **2(lnL(M1)-ln(LM2))** | **lnL** | **Parameters estimated** | **2(lnL(M1)-lnL(M2))** |
| **0 (fixed** ω) | -12474.53391 | κ, ω_0_ | 0 vs 1  ≥152.1 (df = 59)  P ≤3.73e-10 | -11696.50377 | κ, ω_0_ | 0 vs 1  ≥143.1 (df = 53)  P ≤3.18e-10 |
| **1 (one** ω **per branch)** | -12398.24327  -12398.35790  -12398.49126 | κ and ω values |  | -11624.90286  -11624.90290  -11624.93086 | κ and ω values |  |
| **2 strict (positive selection)**  **GSTe5 branch allowed ω_1_ ≥ ** | -12462.75550 | κ, ω_0_, ω_1_ | 0 vs 2 (strict)  23.6 (df = 1)  P = 1.21e-06 | -11683.12807 | κ, ω_0_, ω_1_ | 0 vs 2 (strict)  26.8 (df=1)  P = 2.32e-07 |
| **2 relaxed (relaxed constraint)**  **GSTe5 branch allowed ω_1_ = ** | -12463.57587 | κ, ω_0_ | Strict vs relaxed  1.64 (df = 1)  P = 0.20 (NS) | -11684.34663 | κ, ω_0_ | Strict vs relaxed  2.44 (df = 1)  P = 0.12 (NS) |

df: degrees of freedom, NS: not significant.
