# Supplementary material for: Comparative Genomics of the Anopheline Glutathione S-Transferase Epsilon Cluster
Source: PLoS One. 2011 Dec 19;6(12):e29237. doi: 10.1371/journal.pone.0029237 (PMC3242777; doi:10.1371/journal.pone.0029237)
Supplement: Table S7 — Test of positive selection at sites in the GSTe5 branch: comparison of likelihoods of branch site models. (DOC) [file pone.0029237.s010.doc]

Supplementary Table S7: Test of positive selection at sites in the GSTe5 branch: comparison of likelihoods of branch site models

| Dataset | Model | lnL | 2(lnL(M1)-lnL(M2)) |
| --- | --- | --- | --- |
| GST all | A_1_ (relaxed constraint) | -12426.48043 | 26.2 P = 3.05e-07 |
|  | A_2_ (positive selection) | -12413.36925 |  |
| GST no e6 pfd | A_1_ (relaxed constraint) | -11633.87880 | 28.6 P = 8.99e-08 |
|  | A_2_ (positive selection) | -11619.59060 |  |
