# Supplementary material for: Comparative Genomics of the Anopheline Glutathione S-Transferase Epsilon Cluster
Source: PLoS One. 2011 Dec 19;6(12):e29237. doi: 10.1371/journal.pone.0029237 (PMC3242777; doi:10.1371/journal.pone.0029237)
Supplement: Table S8 — Estimated ω values for site classes under branch site models. (DOC) [file pone.0029237.s011.doc]

Supplementary Table S8: Estimated  values for site classes under branch site models

|  |  |  | Site class | | | |
| --- | --- | --- | --- | --- | --- | --- |
| Dataset | Model |  | 0 | 1 | 2a | 2b |
| GSTall | A_1_ | p | 0.67894 | 0.02158 | 0.29025 | 0.00923 |
|  |  | Background w | 0.07865 | 1 | 0.07865 | 1 |
|  |  | Foreground w | 0.07865 | 1 | 1 | 1 |
|  | A_2_ | p | 0.77829 | 0.02351 | 0.1924 | 0.00581 |
|  |  | Background w | 0.07866 | 1 | 0.07866 | 1 |
|  |  | Foreground w | 0.07866 | 1 | 999 | 999 |
| GST no e6pfd | A_1_ | p | 0.66526 | 0.0335 | 0.28679 | 0.01444 |
|  |  | Background w | 0.07846 | 1 | 0.07846 | 1 |
|  |  | Foreground w | 0.07846 | 1 | 1 | 1 |
|  | A_2_ | p | 0.76443 | 0.03814 | 0.18805 | 0.00938 |
|  |  | Background w | 0.07829 | 1 | 0.07829 | 1 |
|  |  | Foreground w | 0.07829 | 1 | 999 | 999 |

Footnote: proportion of sites and calculated ω in each site class. 999 is the upper bound for the foreground ω and in this case is due to there having been non synonymous substitutions but no synonymous ones at the sites in that category: dividing by zero means that the estimated ω would be infinity if not bounded.
