# Supplementary material for: Comparative Genomics of the Anopheline Glutathione S-Transferase Epsilon Cluster
Source: PLoS One. 2011 Dec 19;6(12):e29237. doi: 10.1371/journal.pone.0029237 (PMC3242777; doi:10.1371/journal.pone.0029237)
Supplement: Table S9 — Power and false positive rate of site and branch tests for simulated datasets. (DOC) [file pone.0029237.s012.doc]

Supplementary Table S9: Power and false positive rate of site and branch tests for simulated datasets.

| Model | Branch lengths cf GSTe data | ω_2_ | Percent of LRT significant at P ≤ 0.05 |
| --- | --- | --- | --- |
| A1 | 1 x | 1 | 4% (false positives) |
| A2 | 1 x | 4 | 70% |
| A2 | 1 x | 9 | 92% |
| A2 | 1 x | 999 | 98% |
| A1 | 0.5 x | 1 | 5% (false positives) |
| A2 | 0.5 x | 9 | 83% |
| A1 | 2 x | 1 | 17% (false positives) |
| A2 | 2 x | 9 | 95% |
