# Supplementary material for: Comparative Genomics of the Anopheline Glutathione S-Transferase Epsilon Cluster
Source: PLoS One. 2011 Dec 19;6(12):e29237. doi: 10.1371/journal.pone.0029237 (PMC3242777; doi:10.1371/journal.pone.0029237)
Supplement: Table S10 — Power, accuracy and false positive rate of the BEB method for detecting sites under positive selection in simulated datasets. (DOC) [file pone.0029237.s013.doc]

Supplementary Table S10: Power, accuracy and false positive rate of the BEB method for detecting sites under positive selection in simulated datasets.

| Branch lengths cf GSTe data | ω_2_ | Cut off P value | Cumulative Accuracy | Power | False Positive rate |
| --- | --- | --- | --- | --- | --- |
| 1 x | 4 | >0.950 | 0.935 | 0.241 | 0.004 |
| 1 x | 4 | >0.975 | 0.960 | 0.187 | 0.002 |
| 1 x | 4 | >0.990 | 0.983 | 0.145 | 0.001 |
| 1 x | 9 | >0.950 | 0.942 | 0.322 | 0.005 |
| 1 x | 9 | >0.975 | 0.970 | 0.238 | 0.002 |
| 1 x | 9 | >0.990 | 0.995 | 0.179 | 0.000 |
| 1 x | 999 | >0.950 | 0.983 | 0.516 | 0.002 |
| 1 x | 999 | >0.975 | 0.991 | 0.409 | 0.001 |
| 1 x | 999 | >0.990 | 0.999 | 0.259 | 0.000 |
| 0.5 x | 9 | >0.950 | 0.953 | 0.298 | 0.004 |
| 0.5 x | 9 | >0.975 | 0.966 | 0.192 | 0.002 |
| 0.5 x | 9 | >0.990 | 0.988 | 0.125 | 0.000 |
| 2 x | 9 | >0.950 | 0.943 | 0.324 | 0.005 |
| 2 x | 9 | >0.975 | 0.969 | 0.253 | 0.002 |
| 2 x | 9 | >0.990 | 0.985 | 0.180 | 0.001 |
