# Supplementary material for: Comparative Genomics of the Anopheline Glutathione S-Transferase Epsilon Cluster
Source: PLoS One. 2011 Dec 19;6(12):e29237. doi: 10.1371/journal.pone.0029237 (PMC3242777; doi:10.1371/journal.pone.0029237)
Supplement: Table S11 — Motifs discovered by MEME on the intergenic regions (IR) and 3′UTRs data set. (DOC) [file pone.0029237.s014.doc]

Supplementary Table S11: Motifs discovered by MEME on the intergenic regions (IR) and 3’UTRs data set.

| **Motif identified** | **Specific to** | **P-value** |
| --- | --- | --- |
| 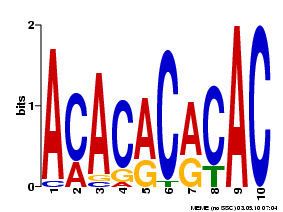 | all IR | From 7.00e-05 to 8.43e-07 |
| 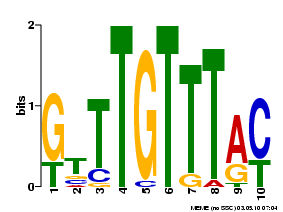 | all IR | From 2.47e-04 to 1.58e-06 |
| 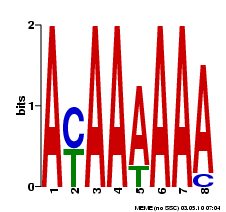 | all IR | From 2.30e-04 to 3.55e-05 |
| 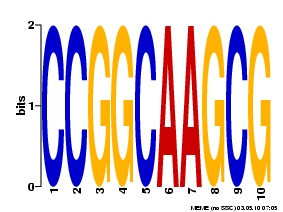 | All IR 4-2 | 3.28e-07 |
| 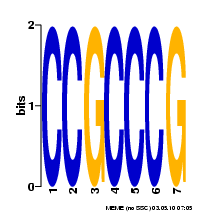 | IR 6-5 for *An. gambiae* and *An. plumbeus* only | 1.86e-05 |
| 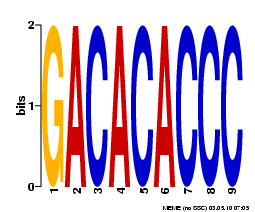 | IR 2-1 for *An. funestus* and *An. stephensi* only | 2.13e-06 |
| 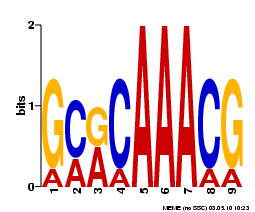 | 3UTR GSTE2 | From 7.63e-05 to 1.88e-06 |
| 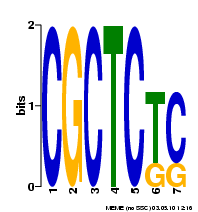 | 3UTR GSTE4 | From 1.80e-04 to 4.83e-05 |
| 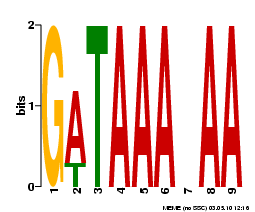 | 3UTR GSTE4 | From 4.61e-05 to1.07e-05 |
| 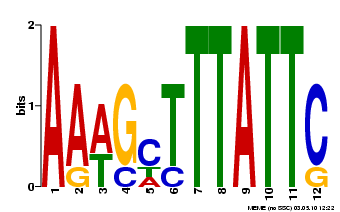 | 3UTR GSTE4 | From 3.47e-06 to 6.70e-08 |
