# Supplementary material for: Comparative Genomics of the Anopheline Glutathione S-Transferase Epsilon Cluster
Source: PLoS One. 2011 Dec 19;6(12):e29237. doi: 10.1371/journal.pone.0029237 (PMC3242777; doi:10.1371/journal.pone.0029237)
Supplement: Table S12 — Predicted targets of An. gambiae miRNAs (aga-mir-X) or D. melanogaster mirRNAs (dme-mir-X) in experimentally determined 3′ UTRs of An. stephensi, An. plumbeus and An. funestus GST genes and sequences 3′ of An. gambiae genes (maximum length 1 kb). Potential miRNA targets were identified using miRanda [48], [49]. (DOC) [file pone.0029237.s015.doc]

Supplementary Table S12: Predicted targets of *An. gambiae* miRNAs (aga-mir-X) or *D. melanogaster* mirRNAs (dme-mir-X) in experimentally determined 3’ UTRs of *An. stephensi*, *An. plumbeus* and *An. funestus* GST genes and sequences 3’ of *An. gambiae* genes (maximum length 1kb). Potential miRNA targets were identified using miRanda [46, 47].

| **Target** | **miRNA** | **Score** | **DeltaG** | ***P*-Value** | **Wobble (W) or mismatch (M) in seed region (2-7)** |
| --- | --- | --- | --- | --- | --- |
| 3UTR5STB | aga-miR-133 | 18.44 | -21.71 | 1.50E-02 | W4 |
| 3UTR5STA | aga-miR-133 | 18.55 | -21.71 | 1.38E-02 | W4 |
| 3UTR5FU | aga-miR-210 | 17.69 | -27.54 | 7.52E-02 | Yes |
| 3UTR1GA | aga-miR-210 | 17.52 | -20.06 | 8.49E-02 | Yes |
| 3UTR6GA | aga-miR-219 | 17.1 | -20.33 | 7.06E-02 | W6 |
| 3UTR1FU | aga-miR-276-5p | 17.03 | -23.6 | 5.04E-02 | W2 |
| 3UTR4GA | aga-miR-278 | 17.75 | -29.59 | 4.88E-02 | Yes |
| 3UTR5GA | aga-miR-9c | 17.71 | -20.69 | 8.20E-02 | W6 |
| 3UTR1ST | aga-miR-989 | 18.52 | -22.31 | 4.60E-02 | Yes |
| 3UTR5STB | aga-miR-375 | 18.44 | -23.51 | 8.65E-02 | W2 |
| 3UTR5STA | aga-miR-375 | 18.55 | -23.51 | 7.99E-02 | W2 |
| RACEPSEUDO1 | aga-miR-263b | 17.39 | -22.17 | 5.13E-02 | Yes |
| 3UTR5PL | dme-miR-3 | 18.23 | -20.94 | 2.31E-02 | W57 |
| 3UTR5FU | dme-miR-284 | 17.83 | -25.78 | 5.78E-02 | W56 |
| 3UTR2GA | dme-miR-284 | 18.04 | -22.52 | 4.99E-02 | Yes |
| 3UTR5GA | dme-miR-34 | 17.65 | -23.12 | 9.51E-02 | Yes |
| 3UTR5FU | dme-miR-210 | 18.13 | -22.41 | 5.89E-02 | Yes |
| 3UTR5GA | dme-miR-9c | 17.71 | -20.69 | 8.20E-02 | W6 |
| 3UTR4PL | dme-miR-310 | 18.2 | -24.28 | 4.55E-02 | W3M5 |
| 3UTR1GA | dme-miR-iab-4as-3p | 18.88 | -26.58 | 4.10E-02 | W4 |
| 3UTR5STB | dme-miR-954 | 18.07 | -26.13 | 5.89E-02 | M5 |
| 3UTR5STA | dme-miR-954 | 18.19 | -26.13 | 5.41E-02 | M5 |
| 3UTR5FU | dme-miR-955 | 18.51 | -25.74 | 3.78E-02 | Yes |
| 3UTR5STA | dme-miR-375 | 18.32 | -21.82 | 9.99E-02 | W2 |
| 3UTR1ST | dme-miR-989 | 17.91 | -21.43 | 6.78E-02 | Yes |
| 3UTR4GA | dme-miR-993 | 16.53 | -21.03 | 7.62E-02 | W4 |
| 3UTR5GA | dme-miR-995 | 18.01 | -23.15 | 4.09E-02 | Yes |
| 3UTR7PL | dme-miR-1003 | 19.45 | -24.67 | 2.23E-02 | W3 |
